# Validation and comparative study of the Motus system for accurately identifying movement behaviours using different sampling frequencies

**DOI:** 10.1038/s41598-025-26373-7

**Published:** 2025-11-27

**Authors:** Tonje Pedersen Ludvigsen, Sebastian Asmussen Sode Hørlück, Jon Roslyng Larsen, Christina Bach Lund, Pasan Hettiarachchi, Mikkel Brandt, Nidhi Gupta

**Affiliations:** 1 National Research Centre for the Working Environment, Copenhagen, Denmark; 2 Department of Occupational and Social Medicine, Holbaek Hospital, Holbaek, Denmark; 3 Department of Medical Sciences, Occupational and Environmental Medicine, Uppsala University, Uppsala, Sweden

**Keywords:** Accelerometry, Wearable, Surveillance, Thigh-worn, SurPASS, Health services, Occupational health, Public health, Behavioural methods, Software

## Abstract

Accurate data on movement behaviours is essential for public health policy and interventions. Accelerometer-based tools like ActiPASS, paired with AxivityX3 accelerometers, are widely used but face challenges like slow data processing and administrative burden. To address these issues, we developed Motus (alias SurPASS), a scalable accelerometer-based system with wireless SENSmotionPlus accelerometers and cloud storage. While Motus shows promising feasibility, validating its performance at different sampling frequencies can further improve battery life, data transfer, and storage. This study aimed to validate Motus in identifying movement behaviours at different sampling frequencies and to compare it with ActiPASS. Data collection included laboratory and free-living sessions, during which participants wore three thigh-mounted accelerometers: SENSmotionPlus (25 Hz and 12.5 Hz) and AxivityX3 (25 Hz). The laboratory session was video recorded and comprised structured and semi-structured movement behaviours, including sedentary, standing, walking, stair climbing, running, and cycling. In the free-living session, participants wore accelerometers for 48 h and carried out usual activities. Data were processed using Motus, with free-living sessions additionally analysed with ActiPASS. The F1-score and balanced accuracy were used to assess Motus performance against video annotations, while Bland-Altman plots evaluated agreement between F1-scores (25 Hz vs. 12.5 Hz) and between Motus (25 Hz and 12.5 Hz) and ActiPASS, with ActiPASS as the reference method. Eighteen participants (61% female, age 34.1 ± 8.3 years) completed the data collection. A total of 395.7 min from the laboratory (mean: 22.0 ± 2.7 min) and 501,122 min from the free-living session (mean: 2638 ± 499.7 min) were analysed. In the laboratory, overall F1-score and balanced accuracy were 0.94 for both frequencies, compared with video observations. Additionally, mean bias F1-scores were ± 0.01 for all behaviours when comparing the performance of Motus 12.5 Hz vs. 25 Hz against videos. In free-living, Motus 25 Hz vs. ActiPASS showed a mean difference of ± 1 min for all behaviours. For Motus 12.5 Hz vs. ActiPASS, the mean difference were ± 1 min for sedentary, stair climbing and cycling, while a mean difference of + 5.1, -2.9 and − 2.2 min were observed for standing, walking and running, respectively. In conclusion, our study provides significant insights into the performance of Motus, highlighting the possibility of reducing the sampling frequency from 25 to 12.5 Hz and still obtaining high performance in classifying different movement behaviours.

**Supplementary Information:**

The online version contains supplementary material available at 10.1038/s41598-025-26373-7.

## Introduction

Large-scale and accurate data on movement behaviours, such as physical activity and sedentary behaviours, are essential for informing effective public health interventions, guiding policy development, and informing evidence-based recommendations^1^.

Wearable devices, such as accelerometers placed on the thigh, hip, or wrist, enable continuous movement behaviour monitoring over multiple days, supported by highly accurate processing software^2^. The Prospective Physical Activity, Sitting and Sleep (ProPASS) consortium^3^ developed ActiPASS^4^, which integrates the Acti4^5,6^ classification algorithm to facilitate offline processing and harmonisation of large-scale accelerometer data across global cohort studies^7^. ActiPASS, the state-of-the-art method, is widely used internationally^8^. However, ActiPASS and similar systems are primarily research-focused, limiting accessibility for non-researchers^4^. Additionally, these systems often experience long data processing delays and impose substantial administrative and participant burdens^9–11^.

In response to these challenges, we have developed the Motus system (alias SurPASS)^12,13^ to eliminate in-person meetings, replace paper-based data collection, and automate processing and participant feedback. Motus consists of (1) an easily attachable accelerometer (SENSmotionPlus) with integrated Bluetooth data-transfer technology, (2) a smartphone app that provides participant-instruction and feedback, and transfers data between accelerometer and cloud storage, (3) back-end infrastructure for cloud storage and processing software, (4) processing software, ActiMotus, built upon the validated MATLAB-Acti4 software, and (5) a web-based application for administrators. Recent studies indicate that Motus is highly feasible for large-scale data collection in occupational and public health surveillance^14,15^. However, its reliance on wireless components and cloud storage means that data resolution and volume significantly affect battery life, storage capacity, and transfer time^16^. Investigating the performance of Motus at lower sampling frequencies is therefore crucial to enhancing feasibility, as it could balance performance with practical constraints such as battery life, transfer time, and storage capacity.

Traditionally, systems using accelerometry employ sampling frequencies between 30 and 100 Hz^17^. Recent research shows that lower frequencies^18–20^, below 30 Hz (as used in systems like Motus and ActiPASS^5–7^, are sufficient for monitoring activities of daily living, which rarely exceed 5–6 Hz^21–23^. In fact, Khusainow et al.^20^, using machine learning-based classifiers, found meaningful classification performance of movement behaviours (accuracy of ≥ 93%), even at sampling frequencies as low as 10 Hz. Similar accuracy has been reported for no-code classification methods using 12.5 Hz^23–25^.

Although Motus currently uses a sampling frequency of 25 Hz, recent evidence suggests that it may be possible to reduce it further without compromising performance, potentially enhancing the feasibility of Motus. Thus, our study aimed to evaluate the performance of the Motus system at various sampling frequencies.

The specific objectives are to:

1. Validate Motus against video observations in identifying movement behaviours at 25 Hz and 12.5 Hz sampling frequencies under laboratory conditions.
2. Compare Motus using 25 Hz and 12.5 Hz sampling frequencies against the commonly used ActiPASS tool in identifying movement behaviours in free-living conditions.

## Method

### ActiMotus and ActiPASS and their key similarities and differences

The processing software for Motus, ActiMotus version 1.1.0^26^, and ActiPASS version 2024.05^4^ are both built upon the validated MATLAB-based Acti4 processing software^5,6,27^, which classifies thigh-worn accelerometry into movement behaviours. Acti4 employs a decision-tree method based on movement intensity and accelerometer orientation relative to gravity. Raw triaxial accelerometry data undergo 4th-order Butterworth low-pass filtering before movement intensity (mean acceleration, standard deviation) and orientation (forward/backward inclination) are calculated in 2-second windows with 1-second overlap. Acti4 then classifies movement behaviours into stationary and active categories. Within the stationary category, it distinguishes between standing and sedentary (including lying and sitting) based on orientation. The active category is further divided into specific activities like walking, running, stair climbing, and cycling, using a combination of movement intensity and accelerometer orientation. Acti4 quantifies the duration spent in each movement behaviour, with classification criteria detailed in previous publications^5,28^. While ActiMotus and ActiPASS derive from Acti4, key differences are outlined in the following section. Both are open-source and publicly available, and collaboration to further develop and apply them in future research is welcomed^4,26^.

#### ActiPASS

ActiPASS^4,29,30^ is designed for batch-processing large volumes of thigh-worn accelerometer data from multiple brands^7^, including the commonly used Axivity AX3. To facilitate large-scale data handling, ActiPASS automatically corrects accelerometer placement errors and applies both device- and individual-specific calibrations using free-living data^5,31^. For this study, ActiPASS is regarded as the most up-to-date and precise implementation of the validated Acti4 algorithms.

#### ActiMotus

To enhance usability and automation, the MATLAB-based Acti4 software has been translated into Python and integrated into the Motus system’s back-end infrastructure as ActiMotus version 1.1.0^26^. The source code is presented in Additional File 1 and available on GitHub (https://github.com/acti-motus/acti-motus). It retains Acti4’s core functionalities but is optimised for the Motus environment. A key distinction lies in data processing: in ActiMotus, raw accelerometry data undergo pre-processing in chunks, where movement intensity and accelerometer orientation features are extracted before classification. This approach optimises computational flow, as classification relies on derived features rather than raw data. In contrast, Acti4 directly accesses raw data during classification.

Furthermore, when the SENSmotionPlus accelerometer was introduced into Acti4, a device-specific adjustment was made to align the standard deviation feature with those of more widely used accelerometers, such as Axivity AX3 and ActiGraph GT3X+, upon which Acti4 was originally based. Because SENSmotionPlus has a smaller dynamic range (see specifications in Additional File 2, Table A1), its standard deviation values during high-intensity movements were slightly reduced, likely due to temporary signal saturation. Since the standard deviation is used to define movement intensity thresholds in the ActiMotus decision tree, a second-order polynomial correction was applied during pre-processing to compensate. The implementation of this correction is available in Additional File 1 and the public GitHub repository (https://github.com/acti-motus/acti-motus).

### Recruitment and ethics

Participants were recruited from a midsized workplace in Denmark, where most held white-collar jobs. The study aimed to enrol 15–25 participants, a typical sample size in similar validation research^5,6,32,33^. Exclusion criteria included allergies to plaster (to avoid adverse skin reactions), pregnancy, or health conditions that could affect normal movement behaviour, including fever on the day of data collection. These criteria were applied to ensure that the system was evaluated under the population conditions for which it was originally developed and validated^5^. In accordance with the Declaration of Helsinki, participants received written and oral study information before providing written informed consent. Ethical approval was granted by the Committee of Scientific Research Ethics for the Copenhagen Region (j.nr. F-24042892). The study complies with national and international data protection regulations.

### Protocol

#### Instrumentation

Data were collected using accelerometers from two manufacturers: SENSmotionPlus (SENS Innovation ApS, Copenhagen, Denmark) and Axivity AX3 (Axivity Ltd., Newcastle, UK). Motus employs the wireless SENSmotionPlus, while ActiPASS typically utilises accelerometers like the Axivity AX3. Axivity AX3 data were used solely for free-living comparison as part of ActiPASS (objective 2). Each participant wore two SENSmotionPlus accelerometers, set to 12.5 Hz and 25 Hz, and one Axivity AX3, set to 25 Hz.

#### Data collection

**Fig. 1:**
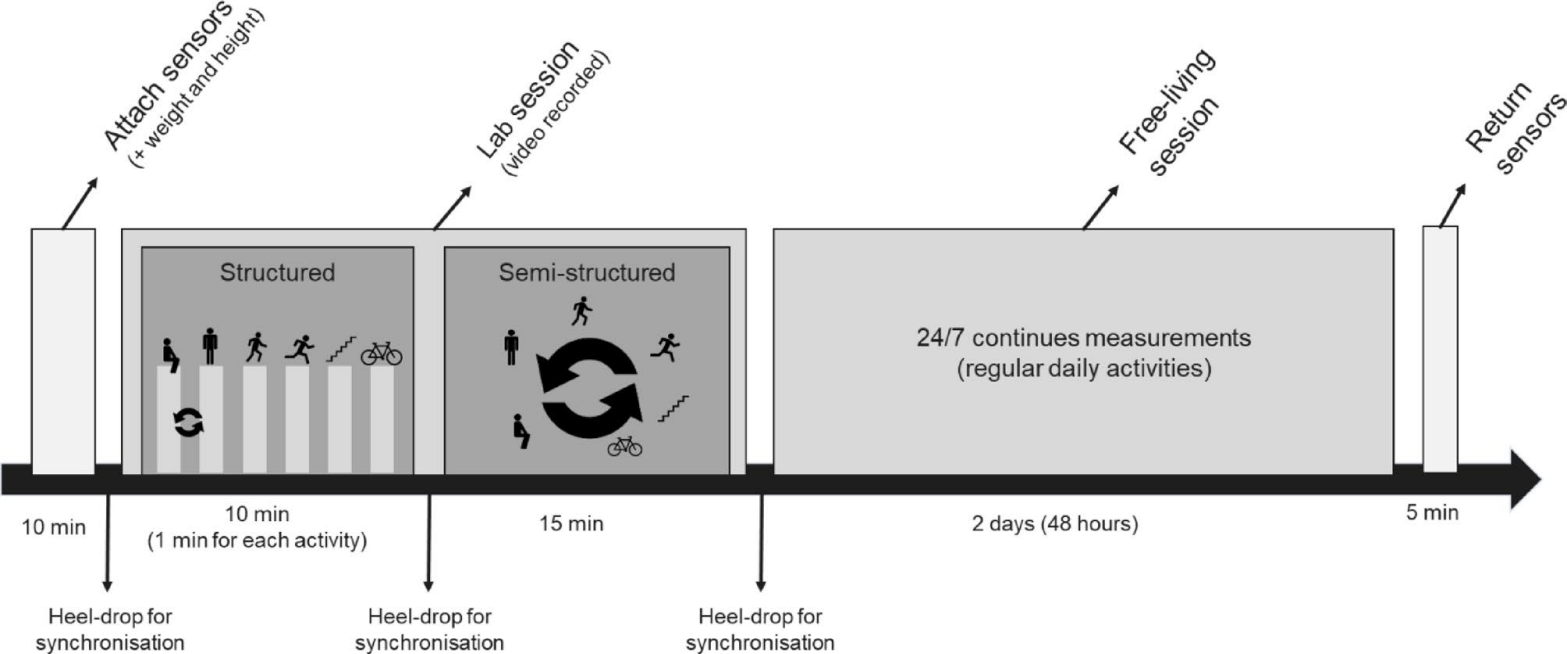
Timeline of data collection, including accelerometer attachment, laboratory and free-living sessions, and device return.

Data were collected over three weeks in May 2023. As shown in Fig. 1, the laboratory session consisted of structured and semi-structured components, followed by a free-living session with continuous measurement over two consecutive days.

#### Preparation

**Fig. 2:**
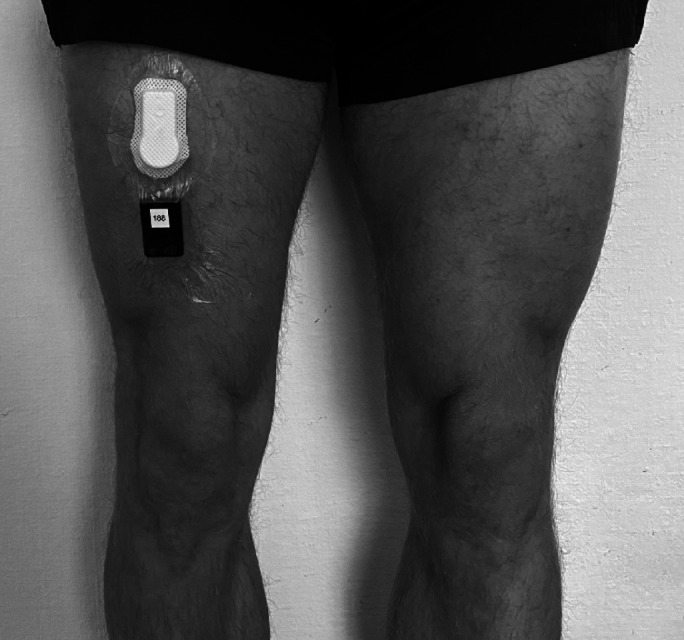
Placement of the accelerometers.

Before the laboratory session, participants’ height and weight were measured. Participants were equipped with three accelerometers placed anteriorly on the midline of the right thigh, specifically at the midpoint between the anterior superior iliac spine and the superior margin of the patella, as visualised in Fig. 2. The two SENSmotionPlus accelerometers were affixed on top of each other under the same plaster, with the order of placement being randomised. The Axivity AX3 was positioned two centimetres above or below the SENSmotionPlus accelerometers, also in a random order.

#### Structured and semi-structured session

In the laboratory, participants completed a session lasting approximately 30 min in indoor and outdoor settings, performing six movement behaviours (sedentary, standing, walking, stair climbing, running, and cycling). They followed a predefined order, beginning with either sedentary (i.e., in the laboratory setting, this means only sitting) or standing to minimise possible order effects and ensure that posture detection was not influenced by always performing one posture before the other. The remaining activities required more setup and were therefore done in a fixed order for practical reasons. Each behaviour lasted one minute, with a 15-second break. The protocol simulated daily activities, e.g., sitting at a computer, walking or running inside and outside. After the structured session, participants engaged in a semi-structured session lasting approximately 15 min, self-selected locations (within the nearby surroundings) and durations for each behaviour. Activities included getting coffee, conversing with colleagues, and cycling in the parking lot. Both sessions were video recorded using a Samsung Galaxy S5 smartphone, held by a research assistant who followed the participant around.

#### Synchronisation

Before, during, and after the laboratory session, participants performed three heel-drops while holding the smartphone camera. These heel-drops produced distinct spikes in the accelerometer signal and corresponding shakes in the video, enabling alignment and synchronisation of accelerometer data across devices.

#### Free-living

After the laboratory session, participants continued wearing all three accelerometers for 48 h during their normal daily routines. Devices were returned after two days. These data were used for a descriptive comparison of the classified time between recordings at different sampling frequencies (12.5 Hz vs. 25 Hz), with ActiPASS used as a reference method (objective 2). This comparison was not intended as a validation but to explore whether and how sampling frequency may lead to differences in classification results under real-world conditions.

### Data Preparation

#### Video annotation

Videos were prepared for manual annotation using the Anvil software package^34^. Video footage was downloaded from the smartphone and converted to AVI format (640 × 360 pixels, 30 fps). Annotation followed a predefined coding scheme describing the onset and offset of movement behaviours (Additional File 3, Table A2). These annotations served as the ground truth for validating movement classifications by Motus at 25 Hz and 12.5 Hz.

Each video was independently annotated twice by separatet raters to assess inter-rater reliability. The inter-rater agreement for classifying movement behaviours was 97%, with Cohen’s kappa (κ = 0.96)^35^.

#### Synchronisation and data processing of accelerometer signal and video

To reduce variability introduced by sensor hardware and individual posture differences, a two-step calibration procedure was performed prior to classification and comparison with video annotations. This included device-specific autocalibration^31^ and individual-specific calibration^36^. This procedure is integrated into both the ActiPASS and Motus systems to harmonise data across devices and minimise inter-monitor variability. To compare classifications from the three accelerometers to video annotations (ground truth), the real-time clocks of the accelerometers and video cameras were synchronised, including alignment and clock-drift correction. Synchronisation was achieved using predefined heel-drop anchor points and signal cross-correlation-based drift correction^37–39^.

For the validation of Motus using 25 Hz and 12.5 Hz sampling frequencies against videos, the following movement behaviours were included: sedentary (sit), standing, walking, running, stair climbing, and cycling. Behaviours categorised as “other,” “invisible,” or “uncertain” by the annotators during the laboratory session were excluded from the analysis. Transitions between behaviours were not annotated as a separate category but were naturally included within the defined activities.

For comparing Motus (25 Hz and 12.5 Hz) with ActiPASS, the same movement behaviours were included, along with lying and moving. Lie was analysed with sit (sedentary), since distinguishing between sitting and lying with a single sensor was beyond this study’s scope and has been validated elsewhere^29^. Move, which refers to brief, irregular activities such as shifting, turning, or taking a few steps, was treated as a transitional state between standing and walking, relevant in free-living conditions. However, it was excluded from the laboratory validation due to the difficulty of reliably staging and annotating such behaviour, as it lacks clear start and end points. Free-living data were averaged over 24 h per participant.

#### Statistical analysis

Statistical analyses were conducted in Python 3.11 using Pandas 1.5^40^ for data wrangling, NumPy 1.24^41^ for various numerical computations, SciPy 1.10^42^ for signal processing and SciKit Learn 1.3^43^ for performance metrics calculation.

##### F1-score and balanced accuracy

System performance was evaluated using F1-score and balanced accuracy for each movement behaviour and overall performance, based on classification data pooled across all participants. These metrics were selected for their widespread use in previous studies, ensuring comparability with past and future research on human activity recognition^5,6,23,33^.

The F1-score for each movement behaviour was calculated as the balanced mean of precision and recall (recall is equivalent to sensitivity). Precision is the proportion of true positives out of all predicted positives, and recall is the proportion of true positives out of all actual positives. In simple terms, precision means how often the system is correct when it says a behaviour is happening, while recall means how often the system detects a behaviour when it actually happens.

The F1-score was then calculated as:

$$\:F{1}_{score}=2\cdot\:\frac{precision\:x\:recall}{precision+recall}$$

The overall F1-score was calculated as a weighted average of the F1-scores for each movement behaviour. The F1-score for each behaviour was weighted based on how often that behaviour occurred in a larger study with 340 participants and 6.5 years of data collected using the Motus system^14^. This approach ensures that the overall score reflects how well the system performs on behaviours that are more likely to happen in real-world situations.

For balanced accuracy, we calculated the mean of recall and specificity for each movement behaviour. Specificity refers to the proportion of true negatives out of all actual negatives. True negatives were identified by a ‘one-against-all’ approach. In simple terms, specificity is how often the system correctly says a behaviour is not happening when it really is not. The balanced accuracy for each behaviour was calculated as the average of recall and specificity:

$$\:Balanced\:accuracy=\frac{recall+specificity}{2}$$

The overall balanced accuracy was calculated as the average of the recall values obtained for each behaviour.

##### Confusion matrix

To assess misclassification trends and classification accuracy, a confusion matrix was generated for each movement behaviour and sampling frequency (25 Hz and 12.5 Hz), as well as for overall system accuracy. Confusion matrices were based on classification data pooled across all participants. These matrices evaluate both the proportion (%) and absolute count of correctly classified instances, highlighting common misclassifications.

##### Bland-Altman plot

The Bland-Altman plot^44^ was used to (a) test the agreement between the F1-scores calculated for different sampling frequencies (25 Hz and 12.5 Hz) and to (b) test the agreement between the minutes spent on various movement behaviours classified by Motus vs. ActiPASS. These analyses were conducted at the participant level, using (a) individual F1-scores and (b) total minutes spent in each movement behaviour. For the agreement between F1-scores, 25 Hz was used as the reference, and for the agreement between Motus and ActiPASS, ActiPASS was used as the reference method and denominator for relative interpretation.

The plot presents the mean bias (mean difference), limits of agreement (mean difference ± 1.96 x standard deviation) and general shape of the spread of data. A mean bias close to zero suggests good agreement. A positive or negative bias indicates whether one method tends to overestimate or underestimate the values compared with the other method. Narrow limits of agreement indicate consistent results between sampling frequencies (F1-score) and systems (minutes), while wider limits suggest greater variability in the results. Regarding the shape of the data spread, a random scatter or no shape indicates consistent differences, while shapes in the scatter indicate proportional biases (e.g., with a higher mean F1-score, the mean difference between two sampling frequencies increases).

## Results

Of the 20 participants who consented to participate, 18 (61% female, age 34.1 ± 8.3 years, body mass index 23.2 ± 2.5 kg/m2) completed the entire protocol with valid data, resulting in 406.2 min of recorded data from the laboratory session (mean ± SD = 22.6 ± 2.6 min) and 50,122 min of recorded data from the free-living session (mean ± SD = 2638 ± 499.7 min). Of the two excluded participants, one was removed due to abnormal movement patterns, and the other due to technical issues with the recorded data.

### Validation of Motus using different sampling frequencies in a laboratory setting

After excluding data annotated and categorised as “other”, invisible”, and “uncertain”, from the lab session, the dataset consisted of 395.7 min. Walking was the dominant activity during the laboratory session, with 154.4 min, while running was the least dominant activity with 29.8 min. The total time spent in sedentary behaviour was 46.7 min, standing 94.4 min, stair climbing 34.2 min, and cycling 36.1 min.

#### Performance metrics

Table 1 shows the performance of Motus using 25 Hz and 12.5 Hz (both SENSmotionPlus) sampling frequencies compared with video observations, in classifying the six targeted movement behaviours (sedentary, standing, walking, running, stair climbing, and cycling). The results demonstrate an F1-score and balanced accuracy of 0.94 for Motus sampling at 25 Hz and 12.5 Hz. Specifically, for Motus sampling at 25 Hz, the F1-score varied from 0.87 (stair climbing) to 0.96 (cycling), and the balanced accuracy varied from 0.95 (sedentary and stair climbing) to 0.99 (running). For Motus sampling at 12.5 Hz, F1-score varied from 0.86 (stair climbing) to 0.96 (standing, walking, running and cycling), and balanced accuracy varied from 0.95 (sedentary and stair climbing) to 0.98 (standing and walking).

**Table 1:** Performance metrics for movement behaviour classification by Motus at 25 Hz and 12.5 Hz.

Sampling frequency	Performance metric	Movement behaviour						
		Sed	Stand	Walk	Run	Stairs	Cycle	Overall
25 Hz	Precision	0.98	0.94	0.96	0.90	0.82	0.98	0.96
	Recall	0.89	0.97	0.94	1.00	0.91	0.94	0.93
	Specificity	1.00	0.98	0.98	0.99	0.98	1.00	0.99
	F1-score	0.93	0.95	0.95	0.94	0.87	0.96	0.94
	Balanced accuracy	0.95	0.97	0.96	0.99	0.95	0.97	0.94
12.5 Hz	Precision	0.98	0.94	0.96	0.96	0.81	0.98	0.96
	Recall	0.89	0.97	0.95	0.96	0.91	0.94	0.92
	Specificity	1.00	0.98	0.98	1.00	0.98	1.00	0.99
	F1-score	0.93	0.96	0.96	0.96	0.86	0.96	0.94
	Balanced accuracy	0.95	0.98	0.96	0.98	0.95	0.97	0.94

Sed = Sedentary (sitting).

#### Confusion matrix

**Fig. 3:**
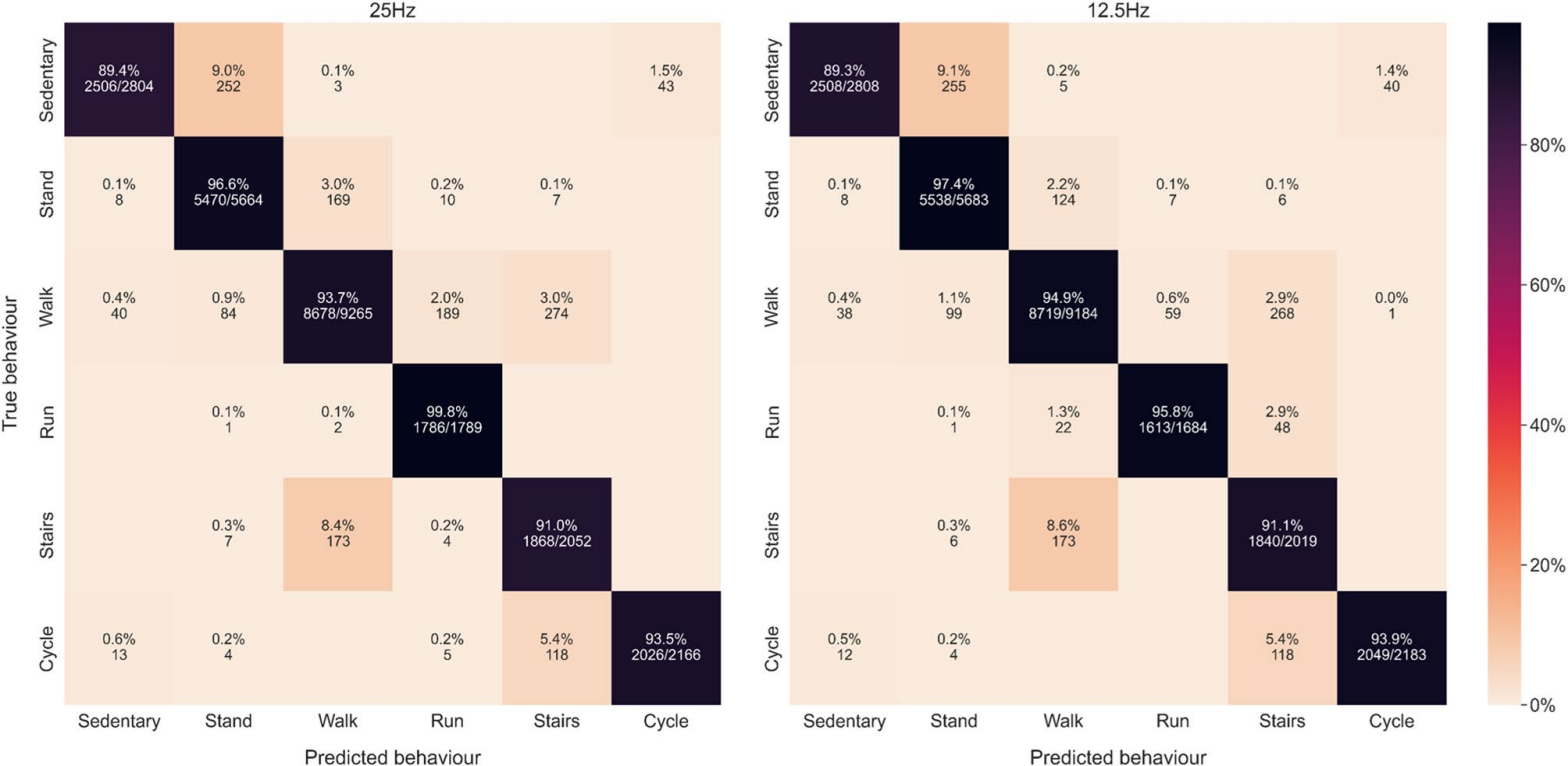
Confusion matrix for Motus using SENSmotionPlus at 25 Hz (left) and 12.5 Hz (right).

Figure 3 shows the confusion matrices, with the diagonal line from the top left to bottom right representing the percentage of correctly classified movement behaviours compared with video annotations for both 25 Hz (right) and 12.5 Hz (left). The overall accuracy was 94% and 95% for 25 Hz and 12.5 Hz, respectively. For specific behaviours, sedentary (~ 89%) and stair climbing (~ 91%) showed the lowest classification accuracy at both frequencies, with most of the sedentary behaviour misclassified as standing (~ 9%) or cycling (~ 2%) and most of the stair climbing behaviour misclassified as walking (~ 9%). For standing, walking and cycling, similar classification accuracy was seen for both sampling frequencies, with an accuracy between ~ 94–97%. The classification accuracy for running was close to 100% for 25 Hz, while for 12.5 Hz, running showed a classification accuracy of ~ 96% with most of the misclassification categorised as walking (~ 3%) or stair climbing(~ 1%).

*The confusion matrix compares the system’s classification of six behaviours (sedentary*,* stand*,* walk*,* run, stairs*,* and cycle) against video observations*,* based on the included sample (n = 18). The Y-axis (rows) represents the true behaviours (based on video annotations)*,* while the X-axis (columns) represents the predicted behaviour (from accelerometer data). Diagonal elements indicate the percentage of correctly classified instances*,* wheras off-diagonal elements represent misclassifications, where one behaviour was incorrectly classified as another.*

#### Bland-Altman plot of agreement between Motus 25 Hz and Motus 12.5 Hz

**Fig. 4:**
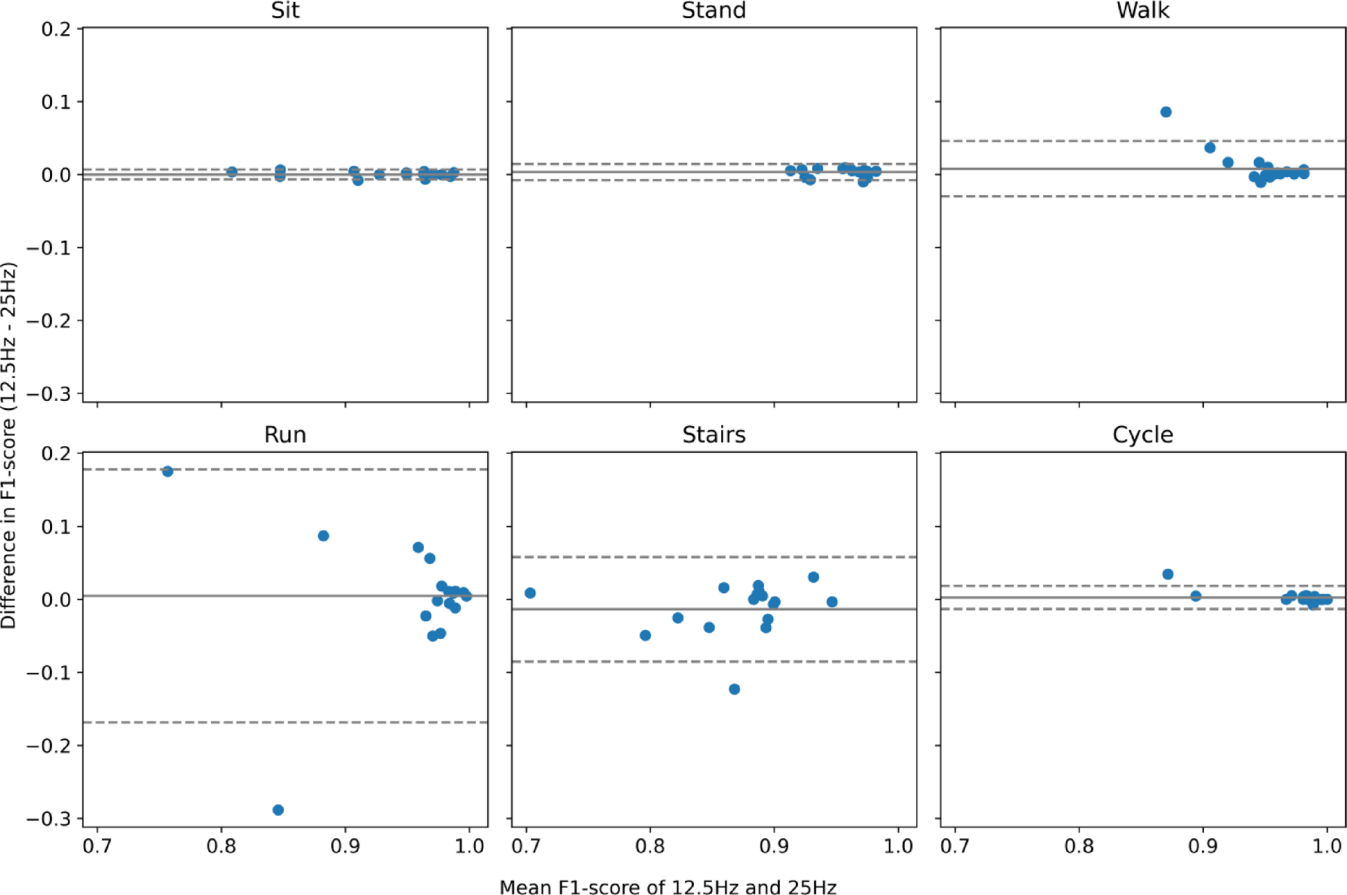
Agreement in F1-Scores for classifying movement behaviours using Motus 25 Hz and 12.5 Hz.

As shown in the Bland-Altman plot, Fig. 4, the mean bias in the F1-score when comparing 12.5 Hz and 25 Hz was close to zero for all behaviours, remaining within ± 0.01 on a scale from 0 to 1. Of the six behaviours analysed, only stair climbing showed a negative mean bias, indicating a lower F1-score at 12.5 Hz compared with 25 Hz. The limits of agreement (dotted lines) were narrowest for sedentary and standing (−0.01 to 0.01), and widest for stair climbing (−0.09 to 0.06) and running (−0.17 to 0.18). For walking, the limits of agreement were − 0.03 to 0.05 and for cycling, −0.01 to 0.02. No specific shape of the data spread was seen for any of the movement behaviours.

*The Bland-Altman plot compares the agreement of F1-scores (scale 0–1) between Motus at two sampling frequencies (25 Hz and 12.5 Hz). The mean bias (mean difference) is presented*,* where a bias close to zero indicates good agreement. A positive bias indicates better performance at 12.5 Hz*,* wheras a negative bias suggests poorer performance. The dashed lines represent the 95% limits of agreement (mean difference ± 1.96 × standard deviation)*,* with narrow limits indicating consistent results and wider limits suggesting greater variability. For visualisation*,* one participant from the cycling behaviour category was removed as an extreme outlier (F1-scores: 0.00 at 25 Hz and 0.03 at 12.5 Hz)*,* potential reasons for this outlier are elaborated in Strengths and limitations.*

### Comparison of motus and ActiPASS during free-living conditions

Table 2 shows the mean minutes and the percentage of total time for each classified movement behaviour, averaged over 24 h, for both Motus (25 Hz and 12.5 Hz) and ActiPASS. Of the six movement behaviours (sedentary, standing, walking, running, stair climbing, and cycling), sedentary was the most dominant behaviour with over 16 h (1098 min), while running was the least dominant behaviour with less than 7 min per day.

**Table 2:** Measured minutes of movement behaviours (Mean ± SD) over 24 h using Motus 25 Hz, Motus 12.5 Hz and ActiPASS.

Movement behaviour	Motus 25 Hz		Motus 12.5 Hz		ActiPASS	
	Mean (± SD)	%	Mean (± SD)	%	Mean (± SD)	%
Sedentary	1098.5 (± 103.8)	76.3	1098.5 (± 103.9)	76.3	1098.6 (± 103.6)	75.7
Stand	162.0 (± 55.7)	11.2	167.5 (± 57.2)	11.6	162.4 (± 56.2)	11.2
Move	61.0 (± 25.2)	4.2	60.8 (± 25.4)	4.2	60.4 (± 25.5)	4.2
Walk	76.6 (± 28.3)	5.3	74.1 (± 27.5)	5.2	76.7 (± 27.7)	5.3
Run	6.1 (± 8.8)	0.4	3.9 (± 7.4)	0.3	6.3 (± 8.8)	0.4
Stairs	8.2 (± 4.7)	0.6	7.6 (± 4.3)	0.5	7.5 (± 3.5)	0.5
Cycle	28.7 (± 22.5)	2.0	28.7 (± 22.5)	2.0	29.2 (± 22.3)	2.0

%Represents the mean percentage of each movement behaviour over 24 h, based on the mean value for each participant. SD = standard deviation.

#### Bland-Altman plot of agreement between motus and ActiPASS

**Fig. 5:**
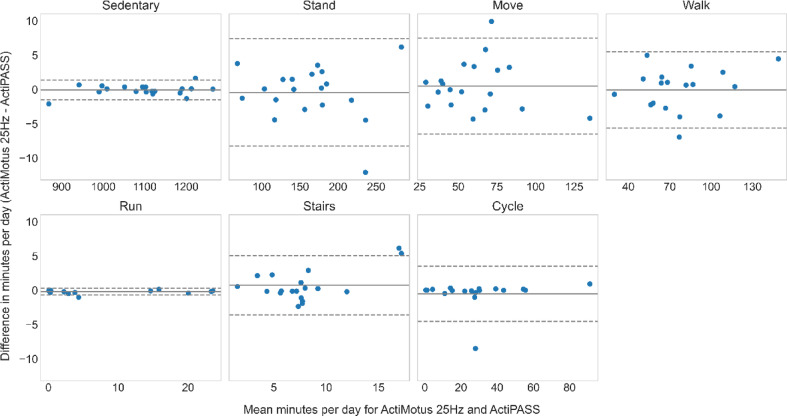
Agreement in movement behaviour classification (minutes) between Motus 25 Hz and ActiPASS.

**Fig. 6:**
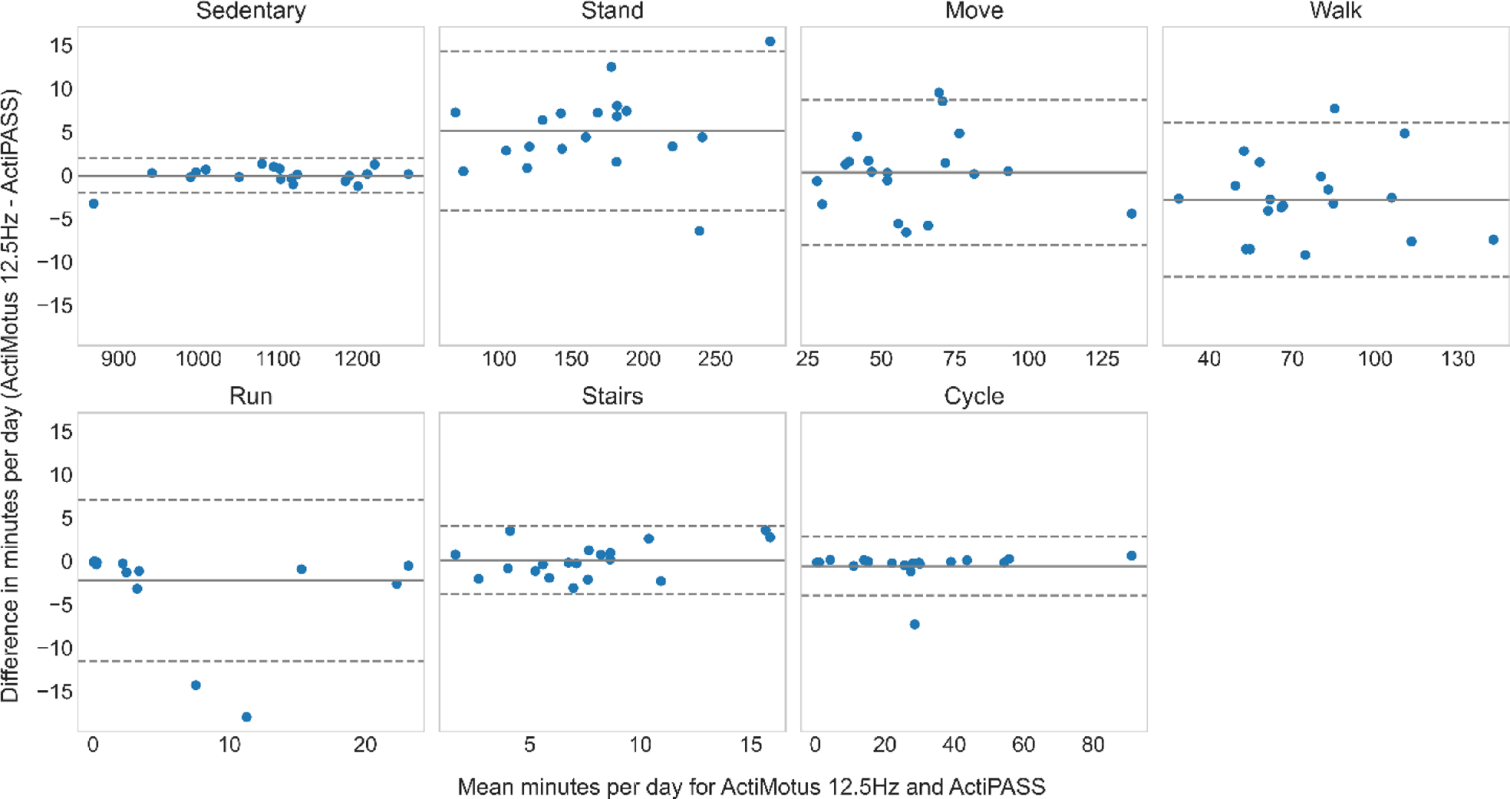
Agreement in movement behaviour classification (minutes) between Motus 12.5 Hz and ActiPASS.

For the free-living data, Bland-Altman plots were used to assess the agreement between the classified minutes for Motus (at 25 Hz and 12.5 Hz) and ActiPASS. Figures 5 and 6 compare differences in absolute time spent on each behaviour for each participant, between Motus 25 Hz and ActiPASS, and Motus 12.5 Hz and ActiPASS, respectively. An overview of the mean bias and limits of agreement in minutes, including relative values based on ActiPASS classifications, is presented in Table 3.

*The Bland-Altman plot compares agreement between Motus (25 Hz) and ActiPASS in estimating minutes spent in each movement behaviour. The mean bias (mean difference) is presented*,* where a bias close to zero indicates good agreement. A positive bias indicates that Motus classified more minutes in a given behaviour than ActiPASS*,* while a negative bias suggests that ActiPASS classified more minutes. The dashed lines represent the 95% limits of agreement (mean difference ± 1.96 × standard deviation)*,* with narrower limits indicating more consistent results and wider limits suggesting greater variability.*

Figure 5 shows the agreement between Motus 25 Hz and ActiPASS, with a mean bias of ± 1 min for all behaviours. Relative to the total time classified for each specific behaviour, this corresponds to ± 1% of the time in sedentary, standing, moving and walking, while for the behaviours running, stair climbing and cycling, the mean bias results in −3%, 10% and − 2% respectively. The narrowest limits of agreement were observed for sedentary (−1.5 to 1.4 min), while the greatest limits of agreement were observed for stair climbing (−3.6 to 5.1 min).

*The Bland-Altman plot compares agreement between Motus (12.5 Hz) and ActiPASS in estimating minutes spent in each movement behaviour. The mean bias (mean difference) is presented*,* where a bias close to zero indicates good agreement. A positive bias indicates that Motus classified more minutes in a given behaviour than ActiPASS*,* wheras a negative bias suggests that ActiPASS classified more minutes. The dashed lines represent the 95% limits of agreement (mean difference ± 1.96 × standard deviation)*,* with narrower limits indicating more consistent results and wider limits suggesting greater variability.*

Figure 6 shows the agreement between Motus 12.5 Hz and ActiPASS with a mean bias of ± 1 min for all behaviours except for standing, walking and running, with mean biases of 5.1 min, −2.9 min and − 2.2 min, respectively. Relative to the total time classified in each specific behaviour, this corresponds to 0%, 1%, 2% and − 2% for sedentary, moving, stair climbing and cycling, while for standing, walking and running, this corresponds to 3%, −4% and − 36%. For limits of agreement, the narrowest limits were observed for sedentary (−2.1 to 2.0 min), while the widest limits were seen for standing (−4.1 to 14.3 min).

No specific trend of the data spread was seen for any of the behaviours when comparing agreement between Motus 25 Hz and ActiPASS (Fig. 5) or Motus 12.5 Hz and ActiPASS (Fig. 6).

**Table 3:** Mean bias and limits of agreement (minutes) for Motus and ActiPASS comparisons.

Movement behaviour	Motus 25 Hz - ActiPASS			Motus 12.5 Hz - ActiPASS		
	Mean bias (%)	LoA-	LoA+	Mean bias (%)	LoA-	LoA+
Sedentary	−0.1 (−0%)	−1.5	1.4	0.0 (0%)	−2.1	2.0
Stand	−0.4 (−0%)	−8.3	7.4	5.1 (3%)	−4.1	14.3
Move	0.5 (1%)	−6.5	7.5	0.3 (1%)	−8.0	8.7
Walk	−0.1 (−0%)	−5.6	5.5	−2.9 (−4%)	−11.7	6.0
Run	−0.2 (−3%)	−0.7	0.3	−2.2 (−36%)	−11.5	7.1
Stairs	0.7 (10%)	−3.6	5.1	0.1 (2%)	−3.8	4.1
Cycle	−0.5 (−2%)	−4.5	3.5	−0.6 (−2%)	−4.0	2.8

Mean bias and limits of agreement (LoA) in minutes for Bland-Altman comparisons of Motus (25 Hz) vs. ActiPASS (Fig. 5) and Motus (12.5 Hz) vs ActiPASS (Fig. 6). Relative values are based on the minutes classified by ActiPASS for each movement behaviour.

## Discussion

This study investigated whether Motus performance could be maintained while reducing its sampling frequency from 25 Hz to 12.5 Hz. Thus, we aimed to validate Motus against video observations under standard and semi-structured laboratory conditions, and to compare Motus with the ActiPASS tool in free-living conditions. The findings indicate that reducing the sampling frequency from 25 Hz to 12.5 Hz does not substantially compromise the performance of Motus. Additionally, both frequencies demonstrated strong agreement in performance (F1-score and balanced accuracy) and against ActiPASS across most behaviours.

These results align with previous studies^16,20,23^ and further support the viability of low-frequency accelerometry for detecting basic movement behaviours in daily living. For example, Lendt et al.^23^reported balanced accuracies ranging from 0.82 to 0.99 for similar movement behaviours, using a sampling frequency as low as 12.5 Hz in both laboratory and free-living conditions. Similarly, Khusainow et al.^20^, tested six sampling frequencies (10–60 Hz) and different machine learning algorithms and found that the classifiers achieved meaningful classification performance (accuracy) for sampling frequency as low as 10 Hz (≥ 93%), with peak performance at 20 Hz for different movement behaviours, and no statistically significant relationship between sampling frequency and classification performance. Moreover, Kahn et al.^16^ investigated optimal sampling frequencies, identifying non-optimal frequencies and emphasising the waste of resources, as higher sampling rates require more battery and storage usage. For example, their results showed that reducing sampling rates from much higher original values (e.g., 96 Hz for quality control activities in manufacturing, 100 Hz for physical activities such as running and cycling, and 250 Hz for walking and gait recognition) to lower ones, such as 32 Hz, 17 Hz, and 12 Hz, led to significant reductions in battery and storage consumption without sacrificing classification performance. Together, these studies highlight the suitability of low sampling frequencies for accelerometer-based classification of human movement behaviours. Additionally, they emphasise that lower sampling frequencies in large-scale surveillance can reduce data transfer time, conserve storage capacity, and extend accelerometer battery life.

Despite this, we did observe some differences between Motus 25 and 12.5 in classifying vigorous activity, such as running. Specifically, 12.5 Hz misclassified running more often than 25 Hz (95.8% vs. 99.8% respectively) in the laboratory, compared with video. We also identified wider limits of agreement for running compared with the other behaviours, suggesting that, for some participants, the difference in F1-score could be larger even though the overall agreement in F1-score was strong. Furthermore, in free-living, Motus 25 and ActiPASS were similar, while Motus 12.5 and ActiPASS showed some differences, with Motus 12.5 Hz classifying 2.2 min less for running compared with ActiPASS. This indicates that Motus 12.5 Hz underestimates running compared with ActiPASS. However, running is a relatively rare behaviour, accounting for 3.9 to 6.3 min of the whole day, resulting in a relatively large underestimation. This underestimation may influence downstream analyses, particularly in studies focusing on physical activity intensity patterns or adherence to guidelines for vigorous activity. As a potential mitigation strategy, we note that such vigorous activity could be combined with similar activity categories (e.g., collapsing running with brisk walking or general moderate to vigorous activity categories) in analytical models, depending on the study purpose.

The potential underestimation of vigorous activities such as running may be explained by to two factors. First, running involves higher frequencies within the human movement range (0.5–10 Hz)^45,46^, and a sampling rate of 12.5 may be too low to capture these accurately without aliasing, as required by the Nyquist–Shannon sampling theorem^16,47^. Second, even if the frequency content is preserved, low sampling rates can miss short, high-amplitude events like foot strikes, leading to underestimation of an variability in the signal. While cadence tends to remain similar across different running speeds, stride length increases, resulting in greater impact forces when the feet hit the ground. Because ActiMotus uses the standard deviation of the signal from these impacts to identify running, the standard deviation may appear lower due to missing high-frequency and/or high-amplitude data, leading to misclassification at 12.5 Hz^5^. Ultimately, the choice of sampling frequency should be guided by the specific research question and the accepted trade-off between the need for data resolution and practical constraints such as battery life and storage capacity.

Besides looking at the differences between the two sampling frequencies, we observed stair climbing to be the movement behaviour with the poorest performance for both sampling frequencies. Stair climbing had the lowest overall performance (F1-score: 0.87 for 25 Hz and 0.86 for 12.5 Hz), indicating a less strong balance between precision and recall compared with the other movement behaviours (≥ 93%). However, the agreement between 12.5 Hz and 25 Hz, as well as ActiPASS, was strong, indicating no systematic under- or overestimations between sampling frequencies or systems. Still, the limits of agreement were wider compared with the other behaviours, indicating that, for some participants, the difference could be larger. This is also seen for similar studies^6,33^ and can be explained by how the algorithm is developed. ActiMotus (the algorithm in Motus) uses a fixed decision tree and calculates an individual threshold for each participant, based on available data, to distinguish walking from stair climbing^5^. Typically, this threshold becomes more precise with larger datasets. However, in this study, each participant had only ~ 20 min of laboratory data, significantly less than the extensive data (e.g., seven days) used in surveillance studies, leading to greater uncertainty. This limited data volume increases uncertainty in threshold estimation, making misclassification more likely. Since stair climbing and walking have similar movement patterns, the key distinguishing factor is the angle of the thigh (sensor placement), specifically, how high the thigh is lifted. If the threshold for detecting stair climbing is set too high, the algorithm may misclassify stair climbing as walking. This is supported by the data, which shows that approximately 8% of stair-climbing was incorrectly classified as walking. Future studies should investigate the sensitivity to the amount of data used for calculating thresholds, as well as how staircase height, sensor placement on the thigh (distance from the knee), and leg length influence classification accuracy.

### Strengths and limitations

One of the notable strengths of our study was the inclusion of two independent annotators, which enhanced both the reliability and validity of the annotated data used for validation reference. Additionally, the validation incorporated data from both structured and semi-structured settings, ensuring applicability to real-life scenarios, with video observations serving as the gold standard for validating movement behaviours. Furthermore, the two-step calibration procedure, including device-specific autocalibration^31^ and individual-specific calibration^36^, minimised potential sensor- and individual-related variability. We also applied a combination of complementary evaluation methods, including confusion matrices, F1-scores, balanced accuracy, and Bland–Altman plots to capture different aspects of system performance. This multi-method approach allows for both straightforward comparisons with existing systems and a more detailed assessment of class-wise performance, systematic differences, and agreement across settings. Finally, the Motus system was compared with an already validated and widely used system in research, ActiPASS, which enhances the reliability and contextual relevance of the results^5,6,33^.

While the free-living comparison was not intended as a validation, the absence of video or other external ground truth still limits the certainty of behaviour classification under real-world conditions. However, capturing free-living activities in a natural environment is extremely time-consuming and presents challenges, not only due to ethical considerations and privacy concerns but also due to the complexity of annotation^48^. The annotation process, which relies on human observation to label activities, can inadvertently misinterpret certain behaviours, further complicating the validation of free-living data. This issue was particularly evident during cycling when the participant was not pedalling, where the accelerometer was positioned on the leg placed on the lowest pedal. Motus identified the accelerometer’s position as “standing,” but the annotators interpreted it as “sedentary,” as they observed the behaviour instead of the accelerometer orientation. This discrepancy highlights the potential for human error in the annotation process, rather than issues with the algorithm itself^48^. While structured procedures were in place and inter-rater agreement was high, further improvements are needed to enhance annotation validity and reduce the risk of shared interpretation bias. In this study, annotators received initial training and followed written protocols, but future work could include a pilot annotation phase with iterative refinement of both procedures and instructional material^49^. This may improve clarity, especially around complex transitions and ambiguous behaviours. Additional accelerometer placements (e.g., on both thighs or the back) could help confirm postural orientation and reduce reliance on visual interpretation alone^30^. Finally, the development of automated annotation tools, potentially using machine learning or artificial intelligence, may also support more scalable and objective labelling in future validation studies.

In addition to annotation-related errors, participant instructions can also introduce misclassification. For instance, during the cycling behaviour, participants were allowed to adjust their seat height freely. However, one participant did not modify the seat after the previous user, resulting in a seat that was too high. Consequently, they were forced to sit on the front edge, altering their leg positioning. As a result, their leg did not reach the angle threshold set by the algorithm for detecting cycling, leading to misclassification. This could potentially explain why all the cycling behaviour for this participant was classified as stair climbing, resulting in poor performance for this participant (F1-score for cycling: 0.00 and 0.03 for 25 Hz and 12.5 Hz, respectively). This issue could have been minimised by ensuring that participants were properly instructed on the importance of a natural seating position. Checking their posture, confirming that they were seated comfortably, and allowing sufficient time for adjustments could have helped reduce such errors. However, it is also possible that such non-optimal cycling postures occur in real-life situations, suggesting that the observed misclassification may reflect a real-world challenge for the system rather than solely a protocol deviation.

### Practical implications and future development

The findings from the present study have important practical implications. Our results show that reducing the sampling frequency does not significantly affect the performance of Motus. This could enhance the overall feasibility of Motus, which has already been demonstrated to be feasible for large-scale data collection^14,15^. However, the feasibility study, conducted in two stages, identified issues in the first stage with time-consuming data transfer, referring to the time required for data to be transmitted from the accelerometer to the smartphone via Bluetooth and subsequent upload to cloud storage. In the second stage of the feasibility trial, when lowering the sampling frequency from 25 Hz to 12.5 Hz, the data transfer time was reduced by approximately 50%. Additionally, by lowering the sampling frequency, the accelerometer generates less data per unit of time, allowing it to store data for longer periods without needing to transfer it to the cloud, thus improving the system’s practical applicability. This suggests that reducing the sampling frequency could be a viable strategy to improve data storage and data transfer efficiency, making the system more suitable for long-term monitoring in real-world applications.

Future development of the Motus system will focus on optimising the performance of Motus at 12.5 Hz and addressing the issues identified in this study. The primary focus is on high-intensity movement behaviours, such as running, as our findings indicate that these behaviours are most affected by reduced sampling frequency. As a result, we have begun refining further adjustments to the correctional polynomial used for the integration of the SENSmotionPlus accelerometer to account for the reduced sampling frequency. A result from this is presented in Additional File 4 and Figure A1. Moving forward, additional refinement of these adjustments, along with testing across various populations, will be crucial. Such development will be open-source and available on GitHub^26^ to facilitate ongoing collaboration within the research community.

### Conclusion

In conclusion, our study provides significant insights into the performance of the Motus system, highlighting the possibility of reducing the sampling frequency from 25 Hz to 12.5 Hz and still obtaining high performance in classifying different movement behaviours. These findings contribute to the existing literature and offer practical solutions for enhancing activity monitoring in real-world settings.

## Supplementary Information

Below is the link to the electronic supplementary material.

Supplementary Material 1

Supplementary Material 2

Supplementary Material 3

Supplementary Material 4

## Data Availability

The data that support the findings of this study can be made available upon reasonable request by contacting the corresponding author at xtpl@nfa.dk. The processing tools ActiPASS ([https://github.com/Ergo-Tools/ActiPASS](https:/github.com/Ergo-Tools/ActiPASS)) and ActiMotus (https://github.com/acti-motus/acti-motus) are publicly available at GitHub.

## Abbreviations

ProPASS: Prospective physical activity and sitting and sleep consortium
SD: Standard deviation
Sed: Sedentary
LoA: Limits of agreement
