# Supplementary material for: Validation and comparative study of the Motus system for accurately identifying movement behaviours using different sampling frequencies
Source: Sci Rep. 2025 Nov 27;15:42377. doi: 10.1038/s41598-025-26373-7 (PMC12661018; doi:10.1038/s41598-025-26373-7)
Supplement: Supplementary file 1 — Supplementary Material 1 [file 41598_2025_26373_MOESM1_ESM.pdf]

Additional file 1: ActiMotus – source code for preprocessing and activity classification in the Motus system

Step 1: Extract features from raw data

```
from typing import Literal

import pandas as pd

from ..utils import get_sampling_frequency
from .calibration import auto_calibrate
from .hl_ratio import get_hl_ratios
from .resample import downsample, upsample
from .steps_features import get_steps_features


def extract_features(
    df: pd.DataFrame,
    gravity_calibration: bool = True,
    sampling_frequency: float | None = None,
    resampling: Literal["legacy", "simple", "poly"] = "legacy",
) -> pd.DataFrame:
    if not sampling_frequency:
        sampling_frequency = get_sampling_frequency(df)

    if sampling_frequency > 30 and resampling != "poly":
        print(
            "Data will be downsampled to 30Hz, therefore we recommend using poly resampling."
        )

    df = upsample(
        df, source_sf=sampling_frequency, type=resampling
    )  # TODO: Rename upsample to resample

    if gravity_calibration:
        df = auto_calibrate(df)

    steps_features = get_steps_features(
        df
    )  # steps_features is calculated on the original data, not on the downsample (with diffrent
    filter and downsampling)
    hl_ratio = get_hl_ratios(
        df
    )  # HL ratio is calculated on the original data, not on the downsample (with diffrent filter
    and downsampling)

    df = downsample(df)
    df["hl_ratio"] = hl_ratio
    df[["walk_feature", "run_feature"]] = steps_features
    df["sf"] = sampling_frequency

    df = df[
        ~df.index.duplicated(keep="first")
    ]  # Remove duplicates indexes after resampling

    return df

import numpy as np
import pandas as pd

from ..utils import butterworth_filtfilt


def _get_low_high(df: pd.DataFrame) -> pd.DataFrame:
    filtered_low = butterworth_filtfilt(df, ["z"], 3, 1, "low")["z"]
    filtered_low.name = "low"
```

```python
    filtered_high = butterworth_filtfilt(df, ["z"], 3, 1, "high")["z"]
    filtered_high.name = "high"

    filtered = pd.concat([filtered_low, filtered_high], axis=1)

    return filtered


def get_hl_ratios(df: pd.DataFrame, window: int = 128) -> pd.Series:
    system_sf = 30
    indexes = df.index[::system_sf].ceil("s")  # type: ignore
    # NOTE: Maybe different window?

    df = _get_low_high(df)
    df[["low", "high"]] = df[["low", "high"]].abs()
    df = (
        df[["low", "high"]]
        .rolling(window, min_periods=1, center=True, step=system_sf)
        .mean()
        .astype(np.float32)
    )
    df["ratio"] = df["high"] / df["low"]
    df.index = indexes

    return df["ratio"]

import numpy as np
import pandas as pd
from sklearn.linear_model import LinearRegression


def _get_valid_indexes(
    series: pd.Series,
    treshold: float,
    seed: int,
    points: int,
) -> list:
    valid_positive_pts = series[(series.notna()) & (series >= treshold)]
    valid_negative_pts = series[(series.notna()) & (series <= -treshold)]

    valid = len(valid_positive_pts) > 1 and len(valid_negative_pts) > 1

    if not valid:
        raise ValueError("No valid data for calibration")

    random_positive_pts = valid_positive_pts.sample(frac=1, random_state=seed).head(
        points
    )
    random_negative_pts = valid_negative_pts.sample(frac=1, random_state=seed).head(
        points
    )

    return random_positive_pts.index.to_list() + random_negative_pts.index.to_list()


def _get_calibration_points(
    df: pd.DataFrame,
    window: int,
    step: int,
    seed: int,
) -> pd.DataFrame:
    n_points = 500
    moving_sd_threshold = 0.013
    valid_pts_threshold = 0.3

    moving_mean = df.rolling(window, 1, True).mean()
    moving_sd = df.rolling(window, 1, True).std()
```

```python
    moving_mean_nth = moving_mean.iloc[::step]
    moving_sd_nth = moving_sd.iloc[::step]

    valid_df = moving_mean_nth[(moving_sd_nth <= moving_sd_threshold).all(axis=1)]

    valid_indexes = []
    for axis in valid_df.columns:
        series = valid_df[axis]
        indexes = _get_valid_indexes(series, valid_pts_threshold, seed, n_points)
        valid_indexes.extend(indexes)

    valid_indexes = pd.to_datetime(valid_indexes).unique()
    valid_df = valid_df.loc[valid_indexes].sort_index()

    return valid_df


def _get_regression_results(df_X, df_y, weights) -> pd.DataFrame:
    results = []
    for axis in df_X.columns:
        regression = LinearRegression().fit(
            df_X[[axis]],
            df_y[[axis]],
            sample_weight=weights,
        )

        scale = regression.coef_[0]
        offset = regression.intercept_

        results.append(
            {
                "axis": axis,
                "scale": scale[0],
                "offset": offset[0],  # type: ignore
            }
        )

    return pd.DataFrame.from_records(results, index="axis")


def auto_calibrate(
    df: pd.DataFrame,
    window: int = 300,
    step: int = 300,
    seed: int = 281_597,
) -> pd.DataFrame:
    max_iter = 1_000
    convergence_treshold = 1e-9
    errors_threshold = 0.02

    try:
        temp = df.copy()
        calibration_pts = _get_calibration_points(temp, window, step, seed)

        calibration_df = calibration_pts.copy()
        calibration_df["weights"] = 1
        variables = pd.DataFrame(
            {"scale": [1, 1, 1], "offset": [0, 0, 0]}, index=["x", "y", "z"]
        )

        for iter in range(max_iter):
            calibration_df[["x", "y", "z"]] = (
                calibration_pts[["x", "y", "z"]]
                .multiply(variables["scale"], axis=1)
                .add(variables["offset"])
            )
```

```python
            calibration_df["vm"] = np.linalg.norm(
                calibration_df[["x", "y", "z"]],
                axis=1,
            )
            calibration_df["errors"] = np.abs(calibration_df["vm"] - 1)

            target_df = calibration_df[["x", "y", "z"]].div(
                calibration_df["vm"], axis=0
            )

            regression = _get_regression_results(
                calibration_df[["x", "y", "z"]], target_df, calibration_df["weights"]
            )

            variables["scale_before"] = variables["scale"].copy()
            variables["scale"] = variables["scale"].multiply(regression["scale"])
            variables["offset"] = variables["offset"].add(regression["offset"])

            calibration_df["weights"] = (1 / calibration_df["errors"]).clip(upper=100)

            convergence_error = sum(abs(variables["scale"] - variables["scale_before"]))

            if convergence_error < convergence_treshold:
                print(f"Convergence achieved after {iter} iterations")
                break

        errors_mean = calibration_df["errors"].mean()

        if errors_mean > errors_threshold:
            print("Calibration not successful.")

        else:
            if iter == max_iter - 1:
                print("No convergence but calibration successful")

            temp = (
                temp[["x", "y", "z"]]
                .multiply(variables["scale"], axis=1)
                .add(variables["offset"], axis=1)
            )
            df = temp

    except ValueError as e:
        print(e)

    return df

from fractions import Fraction
from typing import Literal

import numpy as np
import pandas as pd
from scipy.signal import resample_poly

from ..utils import butterworth_lfilter, get_tensor, hertz_to_timedelta

MATLAB_DATENUM = 719_529


def _get_daily_increment(sf: float) -> float:
    seconds_per_day = 24 * 60 * 60

    return (1 / seconds_per_day) / sf


def _datetime_to_matlab_datenum(
```

```python
    df: pd.DataFrame,
    constant: float,
) -> np.ndarray:
    # TODO: This could be probably done in pandas way with pd.to_datetime to Julian date
    conversion or something similar

    origin_timestamps = (df.index.astype("int64") // 1_000_000).values
    seconds_per_day = 24 * 60 * 60

    return (
        constant + origin_timestamps / 1_000 / seconds_per_day
    )  # This order matters, otherwise the result is little bit of in comparison to the original


def _get_datetime_indexes(
    start: float, end: float, period: int, matlab_datenum: float
) -> pd.DatetimeIndex:
    start_timestamp = start - matlab_datenum
    end_timestamp = end - matlab_datenum

    start_datetime = pd.to_datetime(
        start_timestamp,  # type: ignore
        unit="D",
        utc=True,
    )
    end_datetime = pd.to_datetime(
        end_timestamp,  # type: ignore
        unit="D",
        utc=True,
    )

    datetime_indexes = pd.date_range(
        start_datetime,
        end_datetime,
        period,
        name="datetime",
    )

    return datetime_indexes


def original_upsample(
    df: pd.DataFrame,
    target_sf: float = 30,
) -> pd.DataFrame:
    matlab_timestamps = _datetime_to_matlab_datenum(df, MATLAB_DATENUM)

    start_timestamp = matlab_timestamps[0]
    end_timestamp = matlab_timestamps[-1]

    increment = _get_daily_increment(target_sf)
    resampled_matlab_timestamps = np.arange(start_timestamp, end_timestamp, increment)

    datetime_indexes = _get_datetime_indexes(
        start_timestamp, end_timestamp, len(resampled_matlab_timestamps), MATLAB_DATENUM
    )

    resampled_df = pd.DataFrame(index=datetime_indexes)
    resampled_df["x"] = np.interp(
        resampled_matlab_timestamps, matlab_timestamps, df["x"]
    )
    resampled_df["y"] = np.interp(
        resampled_matlab_timestamps, matlab_timestamps, df["y"]
    )
    resampled_df["z"] = np.interp(
        resampled_matlab_timestamps, matlab_timestamps, df["z"]
    )
```

```python
    n_rows = target_sf * np.fix(len(resampled_df) / target_sf).astype(int)
    resampled_df = resampled_df.head(n_rows)

    return resampled_df


def simplified_upsample(
    df: pd.DataFrame,
    target_sf: float = 30,
) -> pd.DataFrame:
    target_timedelta = hertz_to_timedelta(target_sf)
    start_dt = df.index[0]
    end_dt = df.index[-1]

    datetimes = pd.date_range(
        start_dt,
        end_dt,
        freq=target_timedelta,
        name="datetime",
    )  # Create a new index with the target frequency, i.e. upsample datetimes
    periods = target_sf * np.fix(len(datetimes) / target_sf).astype(
        int
    )  # Get the number of periods to return which are based on full seconds

    df = pd.DataFrame(
        {
            "x": np.interp(datetimes, df.index, df["x"]),
            "y": np.interp(datetimes, df.index, df["y"]),
            "z": np.interp(datetimes, df.index, df["z"]),
        },
        index=datetimes,
    ).head(periods)

    return df


def poly_resample(df: pd.DataFrame, source_sf: float, target_sf: float) -> pd.DataFrame:
    up = Fraction(source_sf)
    down = Fraction(target_sf)

    ratio = Fraction(down, up).limit_denominator()

    print(
        f"Resampling from {source_sf}Hz to {target_sf}Hz with ratio {ratio.numerator}/{ratio.
    denominator}."
    )

    start = df.index[0]
    end = df.index[-1]

    resampled = resample_poly(df[["x", "y", "z"]], ratio.numerator, ratio.denominator)
    resampled = pd.DataFrame(resampled, columns=["x", "y", "z"])
    resampled.index = pd.date_range(start, end, periods=len(resampled), name="datetime")

    periods = target_sf * np.fix(len(resampled) / target_sf).astype(
        int
    )  # Get the number of periods to return which are based on full seconds
    resampled = resampled.head(periods)

    return resampled


def upsample(
    df: pd.DataFrame,
    source_sf: float | None = None,
    target_sf: float = 30,
```

```
    type: Literal["legacy", "simple", "poly"] = "legacy",
) -> pd.DataFrame:
    if source_sf == target_sf:
        n_rows = target_sf * np.fix(len(df) / target_sf).astype(int)
        df = df.head(n_rows)
        print(
            "Source and target sampling frequencies are the same. No resampling is done. Only
    index is cut to the full 30Hz periods."
        )
        return df

    match type:
        case "legacy":
            df = original_upsample(df, target_sf)
        case "simple":
            df = simplified_upsample(df, target_sf)
        case "poly":
            if source_sf is None:
                raise ValueError("source_sf must be provided for poly resampling.")

            df = poly_resample(df, source_sf, target_sf)
        case _:
            raise ValueError(
                f"Unsupported type: {type}. Supported types are 'legacy' and 'simple'."
            )

    return df


def downsample(
    df: pd.DataFrame,
) -> pd.DataFrame:
    system_sf = 30

    df_filtered = butterworth_lfilter(df, 4, 5).astype(np.float32)

    tensor = get_tensor(df_filtered)
    mean = np.mean(tensor, axis=0)
    sd = tensor.std(axis=0, ddof=1)
    sum = np.sum(tensor, axis=0)
    sq_sum = np.sum(np.square(tensor), axis=0)

    resampled_df = np.concatenate([mean, sd, sum, sq_sum], axis=1)
    # TODO: Return only needed features for processing step
    resampled_df = pd.DataFrame(
        resampled_df,
        columns=[
            "x",
            "y",
            "z",
            "sd_x",
            "sd_y",
            "sd_z",
            "sum_x",
            "sum_y",
            "sum_z",
            "sq_sum_x",
            "sq_sum_y",
            "sq_sum_z",
        ],
        index=df.index[::system_sf].ceil("s"),  # type: ignore
    )

    sum_dot_xz = np.sum((tensor[:, :, 0] * tensor[:, :, 2]), axis=0)
    resampled_df["sum_dot_xz"] = sum_dot_xz

    return resampled_df
```

```python
import numpy as np
import pandas as pd
from scipy.fft import fft
from scipy.signal import butter, detrend, lfilter


def _get_filtered_signal(df: pd.DataFrame, sf: float) -> pd.DataFrame:
    b, a = butter(6, 2.5 / (sf / 2), "low")
    x = lfilter(b, a, df["x"], axis=0)

    del b, a

    walk_b, walk_a = butter(6, 1.5 / (sf / 2), "high")
    walk_x = lfilter(walk_b, walk_a, x, axis=0)  # type: np.ndarray # type: ignore

    del x, walk_b, walk_a

    run_b, run_a = butter(6, 3 / (sf / 2), "high")
    run_x = lfilter(run_b, run_a, walk_x)  # type: np.ndarray # type: ignore

    del run_b, run_a

    df = pd.DataFrame({"walk": walk_x, "run": run_x})

    return df


def _get_steps(x: np.ndarray) -> int:
    x = detrend(x)  # type: ignore
    x = fft(x, 512)  # type: ignore
    x = 2 * abs(x[:256])
    x = np.argmax(x)  # type: ignore

    return x  # type: ignore


def get_steps_features(
    df: pd.DataFrame,
    window: int = 128,
) -> pd.DataFrame:
    system_sf = 30
    indexes = df.index[::system_sf].ceil("s")  # type: ignore

    df = _get_filtered_signal(df, system_sf)
    df = (
        df.rolling(window, min_periods=1, center=True, step=system_sf)
        .apply(lambda x: _get_steps(x), raw=True)
        .astype(np.uint8)
    )
    # NOTE: This could be really speed it up by calculating steps only for movement data and not
    on all windows or by using numba, maybe.
    df.index = indexes

    return df

import operator
from datetime import timedelta
from typing import Any, Callable

import numpy as np
import pandas as pd
from scipy.signal import butter, filtfilt, lfilter, medfilt

ACTIVITIES = {
    0: "non_wear",
    1: "lie",
    2: "sit",
```

```
    3: "stand",
    4: "move",
    5: "walk",
    6: "run",
    7: "stairs",
    8: "bicycle",
    9: "row",
    10: "kneel",
    11: "squat",
}


def timedelta_to_hertz(x: timedelta) -> float:
    return 1 / x.total_seconds()


def hertz_to_timedelta(frequency: float) -> timedelta:
    return timedelta(seconds=1 / frequency)


def get_sampling_frequency(
    df: pd.DataFrame,
    samples: int | None = 10_000,
    allowed_frequencies: list[float] = [12.5, 25, 30, 100],
) -> float:
    if samples:
        df = get_consecutive_samples(df, samples)

    timedeltas = pd.Series(df.index.diff())  # type: ignore
    most_frequent_timedelta = timedeltas.mode()

    if len(most_frequent_timedelta) > 1:
        raise ValueError(
            f"Multiple most frequent timedeltas found (seconds): {most_frequent_timedelta.to_list
    ()}"
        )

    detected_sf = timedelta_to_hertz(most_frequent_timedelta[0])

    if allowed_frequencies is None:
        return detected_sf

    for sf in allowed_frequencies:
        if np.isclose(detected_sf, sf, atol=2.5):
            print(
                f"Detected sampling frequency: {detected_sf:.2f}     {float(sf):.2f} Hz"
            )
            return sf  # type: ignore
    else:
        raise ValueError(
            f"Detected sampling frequency ({detected_sf:.2f}) is not allowed"
        )


def get_consecutive_samples(
    df: pd.DataFrame,
    samples: int,
) -> pd.DataFrame:
    if samples < len(df):
        start_row = np.random.randint(len(df))
        df = df.iloc[start_row : start_row + samples]

    return df


def get_tensor(
    df: pd.DataFrame,
```

```python
) -> np.ndarray:
    system_sf = 30

    padded_before = np.concatenate((df[:system_sf], df))
    padded_after = np.concatenate((df, df[-system_sf:]))

    tensor_shape = (system_sf, -1, 3)
    tensor = np.concatenate(
        (
            np.reshape(padded_before, tensor_shape, order="F"),  # type: ignore
            np.reshape(padded_after, tensor_shape, order="F"),  # type: ignore
        ),
        axis=0,
    )
    tensor = tensor[:, 0:-1, :]

    return tensor


def butterworth_filtfilt(
    df: pd.DataFrame,
    columns: list[str],
    order: int | float,
    cut_off,
    filter_type: str,
) -> pd.DataFrame:
    system_sf = 30

    b, a = butter(order, cut_off / (system_sf / 2), filter_type)
    df_filtered = filtfilt(b, a, df[columns], axis=0).astype(np.float32)  # type: ignore
    df_filtered = pd.DataFrame(df_filtered, columns=columns, index=df.index)

    return df_filtered


def butterworth_lfilter(
    df: pd.DataFrame,
    order: int | float,
    cut_off,
    sf: float = 30,
) -> pd.DataFrame:
    b, a = butter(order, cut_off / (sf / 2), "low")
    df_filtered = lfilter(b, a, df[["x", "y", "z"]], axis=0).astype(np.float32)  # type: ignore
    df_filtered = pd.DataFrame(df_filtered, columns=["x", "y", "z"], index=df.index)

    return df_filtered


def get_filtered_activity(
    valid: pd.Series,
    bouts_length: int,
) -> pd.Series:
    length = 2 * bouts_length - 1
    filtered = medfilt(valid.astype(int), length)
    filtered = medfilt(filtered, length)

    valid = pd.Series(filtered.astype(bool), index=valid.index, name="valid")

    return valid


def get_operator(comparison: str) -> Callable[[Any, Any], bool]:
    match comparison:
        case "<":
            return operator.lt
        case "<=":
            return operator.le
```

```python
        case ">":
            return operator.gt
        case ">=":
            return operator.ge
        case "!=":
            return operator.ne
        case "==":
            return operator.eq
        case _:
            raise ValueError(f"Comparison operator {comparison} not supported.")


def get_short_bouts_ids(
    df: pd.DataFrame,
    activity: str,
    bouts: int,
    comparison: str,  # type: ignore
) -> np.ndarray:
    comparison = get_operator(comparison)  # type: Callable[[Any, Any], bool]

    df = df.loc[df["activity"] == activity]
    short_bouts_df = df.groupby("bout_id").filter(lambda x: comparison(len(x), bouts))
    ids = short_bouts_df["bout_id"].unique()

    return ids


def get_angles(
    df: pd.DataFrame,
    euclidean_distance: pd.Series | None = None,
) -> pd.DataFrame:
    angles = pd.DataFrame(index=df.index)

    if isinstance(euclidean_distance, pd.Series):
        angles["euclidean_distance"] = euclidean_distance
        del euclidean_distance
    else:
        angles["euclidean_distance"] = np.linalg.norm(df[["x", "y", "z"]], axis=1)
        angles.loc[angles["euclidean_distance"] == 0, "euclidean_distance"] = np.nan

    angles["inclination"] = np.arccos(
        df["x"] / angles["euclidean_distance"]
    )  # (0-180 degrees), based on x-axis
    angles["side_tilt"] = -np.arcsin(
        df["y"] / angles["euclidean_distance"]
    )  # Sideways tilt, based on y-axis (originally V)
    angles["direction"] = -np.arcsin(
        df["z"] / angles["euclidean_distance"]
    )  # Forward/Backward direction (+-90 degrees), based on z-axis (originally U)

    angles[["inclination", "side_tilt", "direction"]] = angles[
        ["inclination", "side_tilt", "direction"]
    ].apply(np.degrees)

    return angles.astype(np.float32)


def _get_half_indexes(df: pd.DataFrame, bout_id: int) -> tuple[pd.Index, pd.Index]:
    df = df.loc[df["bout_id"] == bout_id, "activity"]  # type: ignore
    middle, odd = divmod(len(df), 2)

    if odd == 1:
        first_half = df.iloc[: middle + 1]
        second_half = df.iloc[middle + 1 :]
    else:
        first_half = df.iloc[:middle]
        second_half = df.iloc[middle:]
```

```python
    return first_half.index, second_half.index


def fix_bouts(
    df: pd.DataFrame,
    activity: str,
    bouts: int,
) -> pd.Series:
    # NOTE: This can be probably speeded up.
    df = (
        df[["activity"]].copy().reset_index(drop=False)
    )  # With int index is much faster than with DatetimeIndex
    df["bout_id"] = (df["activity"] != df["activity"].shift()).cumsum()

    ids = get_short_bouts_ids(
        df, activity, bouts, "<="
    )  # FIXME: I don’t like this one, it should be just less than probably.

    for bout in ids:
        activity_before = df.loc[df["bout_id"] == bout - 1, "activity"]  # type: pd.DataFrame #
    type: ignore
        activity_after = df.loc[df["bout_id"] == bout + 1, "activity"]  # type: pd.DataFrame #
    type: ignore

        if not activity_before.empty and not activity_after.empty:
            first_half, second_half = _get_half_indexes(df, bout)

            df.loc[first_half, "activity"] = activity_before.values[0]
            df.loc[second_half, "activity"] = activity_after.values[0]

        elif not activity_before.empty:
            df.loc[df["bout_id"] == bout, "activity"] = activity_before.values[0]

        else:
            df.loc[df["bout_id"] == bout, "activity"] = activity_after.values[0]

    df.set_index("datetime", drop=True, inplace=True)

    return df["activity"]


def map_activities(
    series: pd.Series,
    input: str = "text",
    categories: dict[int, Any] = ACTIVITIES,
) -> pd.Series:
    match input:
        case "text":
            code_to_activity = {v: k for k, v in categories.items()}
            series = series.map(code_to_activity)
        case "numeric":
            series = series.map(categories)
        case _:
            raise ValueError("Invalid input type. Choose between ’text’ and ’numeric’.")

    if series.isna().all():
        raise ValueError("Probably wrong input type. Please check the input type.")

    return series
```

Step 2: Classify activities from features

```python
from typing import Any

import pandas as pd
```

```python
from .arm import get_inclination
from .calf.activity import get_activity as get_calf_activity
from .thigh.activity import get_activity as get_thigh_activity
from .trunk.activity import get_activity as get_trunk_activity


def detect_activities(
    thigh: pd.DataFrame,
    *,
    trunk: pd.DataFrame | None = None,
    calf: pd.DataFrame | None = None,
    arm: pd.DataFrame | None = None,
    thigh_rotate: bool = False,
    sensor: str = "sens",
    reference_angles: dict[str, Any] | None = None,
    calibrations: list[dict[str, Any]] | None = None,
) -> tuple[pd.DataFrame, dict[str, Any]]:
    activity = pd.DataFrame()

    reference_angles = reference_angles or {}
    last_angles = {}

    if not thigh.empty:
        activity, last_angles["thigh"] = get_thigh_activity(
            thigh, reference_angles.get("thigh"), calibrations, sensor, thigh_rotate
        )
        print("Thigh activity detected.")

        if isinstance(trunk, pd.DataFrame) and not trunk.empty:
            activity, last_angles["trunk"] = get_trunk_activity(
                trunk, activity, reference_angles.get("trunk"), calibrations
            )
            del trunk
            print("Trunk activity detected.")
        else:
            print("No trunk data. Skipping trunk activity detection.")

        if isinstance(calf, pd.DataFrame) and not calf.empty:
            activity = get_calf_activity(calf, activity, thigh)
            del calf, thigh
            print("Calf activity detected.")
        else:
            print("No calf data. Skipping calf activity detection.")

        if isinstance(arm, pd.DataFrame):
            arm_inclination = get_inclination(arm)
            activity = activity.join(arm_inclination, how="left")
            del arm, arm_inclination
            print("Arm inclination detected.")
        else:
            print("No arm data. Skipping arm activity detection.")

    else:
        print("No thigh data. Activity detection skipped.")

    return activity, last_angles

from typing import Any

import numpy as np
import pandas as pd

from ...utils import fix_bouts, get_angles
from ..flip import fix_flipped_sensor, rotate_by_90_degrees_over_x
from ..non_wear import get_non_wear
from ..reference_angle import calculate_angles, get_intervals, get_last_angle
from .activities import (
```

```python
    get_bicycle,
    get_lie,
    get_row,
    get_run,
    get_sit,
    get_stairs,
    get_stand,
    get_walk,
)
from .reference_angle import get_reference_angle, rotate_by_reference_angle
from .steps import get_steps


def _downsample_sd_correction_for_sens(sd: pd.Series, sf: float) -> pd.Series:
    if sf == 25:
        sd = 0.18 * sd**2 + 1.03 * sd
    elif 12.5:
        sd = 0.18 * sd**2 + 1.03 * sd
    else:
        raise ValueError(f"Unsupported sampling frequency: {sf}")

    return sd


def _fix_activities_bouts(df: pd.DataFrame, bouts: dict[str, int]) -> pd.Series:
    activity = df[["activity"]].copy()

    # Order matters here - move position changed
    for category in [
        "row",
        "bicycle",
        "stairs",
        "run",
        "walk",
        "move",
        "stand",
        "sit",
    ]:
        activity["activity"] = fix_bouts(activity, category, bouts[category])

    return activity["activity"]


def _get_activity_column(df: pd.DataFrame) -> pd.Series:
    # Order matters here
    categories = [
        "row",
        "bicycle",
        "stairs",
        "run",
        "walk",
        "stand",
        "sit",
    ]

    df = df[categories].copy()
    df["move"] = True  # If nothing else, it is move

    activity = df[categories + ["move"]].idxmax(axis=1)
    activity = activity.astype(
        pd.CategoricalDtype(categories=categories + ["move", "lie", "non_wear"])
    )
    activity.name = "activity"

    return activity
```

```
def get_activity(
    df: pd.DataFrame,
    reference_angle: dict[str, Any] | None = None,
    calibrations: list[dict[str, Any]] | None = None,
    sensor: str = "sens",
    rotate: bool = False,
) -> tuple[pd.DataFrame, float | None]:
    df = df.copy()  # TODO: Copy only what is needed.
    sf = df["sf"].mode().values[0].item()

    if rotate:
        df = rotate_by_90_degrees_over_x(df)

    df[["euclidean_distance", "inclination", "side_tilt", "direction"]] = get_angles(df)

    df = fix_flipped_sensor(df, "thigh")  # TODO: This should be run each interval.

    non_wear = get_non_wear(df)
    intervals = get_intervals(df, non_wear, reference_angle, calibrations)
    # NOTE: This is the line where flipping should be done on iterating over intervals.

    intervals = calculate_angles(df, intervals, get_reference_angle)
    last_angle = get_last_angle(
        intervals
    )  # TODO: Should always return if something exists and then it would be filtered out if it is
     automatic or not, in next chunk.

    for interval in intervals.to_dict(orient="records"):
        start = interval["start"]
        end = interval["end"]
        mode = interval["mode"]
        angle = interval["angle"]
        wear = interval["wear"]

        start_calibration = interval.get("calibration_start")
        if not start_calibration:
            start_calibration = start

        end_calibration = interval.get("calibration_end")
        if not end_calibration:
            end_calibration = end

        calculation = interval.get("calculation")
        if not calculation:
            calculation = ""

        print(f"THIGH: Interval: {start} - {end} | Mode: {mode} | Wear: {wear}")
        print(f"Calculation: {start_calibration} - {end_calibration} | {calculation}")
        print(f"Angle: {angle}")
        print("---------------------")
        df.loc[start:end] = rotate_by_reference_angle(df[start:end], angle)

    if sensor == "sens":
        df[["sd_x", "sd_y", "sd_z"]] = df[["sd_x", "sd_y", "sd_z"]].apply(
            lambda x: _downsample_sd_correction_for_sens(x, sf)
        )

    bouts = {
        "sit": 5,
        "stand": 2,
        "move": 2,
        "walk": 2,
        "run": 2,
        "stairs": 5,
        "bicycle": 15,
        "row": 15,
        "lie": 1,
```

```python
        "short_off": 600,
        "long_off": 5_400,
        "on": 60,
    }

    df["row"] = get_row(df, bouts_length=bouts["row"])
    df["bicycle"] = get_bicycle(df, bouts_length=bouts["bicycle"])
    df["stairs"], stairs_threshold = get_stairs(df, bouts_length=bouts["stairs"])
    df["run"] = get_run(df, bouts_length=bouts["run"])
    df["walk"] = get_walk(df, stairs_threshold, bouts_length=bouts["walk"])
    df["stand"] = get_stand(df, bouts_length=bouts["stand"])
    df["sit"] = get_sit(df, bouts_length=bouts["sit"])

    df["activity"] = _get_activity_column(df)
    df["activity"] = _fix_activities_bouts(df, bouts)

    df["lie"] = get_lie(df, bouts_length=bouts["lie"])
    df.loc[df["lie"], "activity"] = "lie"

    for category in ["sit", "lie"]:
        df["activity"] = fix_bouts(df, category, bouts[category])

    df.loc[non_wear, "activity"] = "non_wear"
    del non_wear

    df["steps"] = get_steps(df)
    df.loc[(df["activity"] == "walk") & (df["steps"] > 2.5), "activity"] = "run"
    for category in ["run", "walk"]:
        df["activity"] = fix_bouts(df, category, bouts[category])

    df.loc[df["activity"] == "non_wear", "direction"] = (
        np.nan
    )  # NOTE: Should be direction angle removed if non-wear?

    df.rename(columns={"direction": "thigh_direction"}, inplace=True)

    # NOTE: Remove this line, only for testing.
    # last_angle = intervals.iloc[-1].to_dict()

    return df[["activity", "steps", "thigh_direction"]], last_angle

import pandas as pd

from ....utils import get_filtered_activity


def get_sit(
    df: pd.DataFrame,
    stationary_threshold: float = 45,
    bouts_length: int = 5,
) -> pd.Series:
    valid = df["inclination"] > stationary_threshold
    valid = get_filtered_activity(valid, bouts_length)
    valid.name = "sit"

    return valid

import numpy as np
import pandas as pd

from ....utils import get_short_bouts_ids


def _get_thigh_angle(df: pd.DataFrame) -> pd.Series:
    thigh_angle = np.arcsin(df["y"] / np.sqrt(np.square(df["y"]) + np.square(df["z"])))
    thigh_angle = np.degrees(thigh_angle).abs()  # type: ignore
```

```python
    return thigh_angle


def _get_rotational_crossing_points(
    df: pd.DataFrame,
) -> None:
    angle_h_treshhold = 65
    angle_l_treshhold = 64
    noise_margin = 0.05

    df["thigh_angle"] = _get_thigh_angle(df)
    df["low"] = (df["thigh_angle"] <= angle_l_treshhold).diff()
    df["high"] = (df["thigh_angle"] >= angle_h_treshhold).diff()

    df["noise"] = df["thigh_angle"].diff().abs()
    df["noise"] = df["noise"] >= noise_margin

    df["low"] = df["low"] & df["noise"]
    df["high"] = df["high"] & df["noise"]


def get_lie(
    df: pd.DataFrame,
    bouts_length: int,
) -> pd.Series:
    df = df[["activity", "y", "z"]].copy()

    df["lie"] = False
    df["bout_id"] = (df["activity"] != df["activity"].shift()).cumsum()

    _get_rotational_crossing_points(df)

    short_bouts = get_short_bouts_ids(
        df, "sit", bouts_length, ">"
    )  # NOTE: Activity "sit" needs to be already in the activities dataframe.

    for bout in short_bouts:
        specific_bout = df.loc[df["bout_id"] == bout]
        low = specific_bout["low"].any()
        high = specific_bout["high"].any()

        if low and high:
            df.loc[specific_bout.index, "lie"] = True

    return df["lie"]

import numpy as np
import pandas as pd

from ....utils import get_filtered_activity


def get_stand(
    df: pd.DataFrame,
    stationary_threshold: float = 45,
    move_threshold: float = 0.1,
    bouts_length: int = 2,
) -> pd.Series:
    sd_max = np.max(df[["sd_x", "sd_y", "sd_z"]], axis=1)
    valid = (df["inclination"] < stationary_threshold) & (sd_max < move_threshold)

    valid = get_filtered_activity(valid, bouts_length)
    valid.name = "stand"

    return valid
```

```python
import pandas as pd

from ....utils import get_filtered_activity


def get_run(
    df: pd.DataFrame,
    stationary_threshold: float = 45,
    run_threshold: float = 0.72,
    bouts_length: int = 2,
) -> pd.Series:
    valid = (df["sd_x"] > run_threshold) & (df["inclination"] < stationary_threshold)

    valid = get_filtered_activity(valid, bouts_length)
    valid.name = "run"

    return valid

import pandas as pd

from ....utils import get_filtered_activity


def get_row(
    df: pd.DataFrame,
    move_threshold: float = 0.1,
    bouts_length: int = 15,
) -> pd.Series:
    valid = (90 < df["inclination"]) & (move_threshold < df["sd_x"])
    valid = get_filtered_activity(valid, bouts_length)
    valid.name = "row"

    return valid

import numpy as np
import pandas as pd

from ....utils import get_filtered_activity


def _get_stairs_threshold(
    df: pd.DataFrame,
    run_threshold: float,
) -> float:
    threshold = 4

    valid = df["sd_x"].between(0.25, run_threshold, inclusive="neither") & (
        df["direction"] < 25
    )

    stairs_threshold = df[valid]["direction"]
    stairs_threshold = threshold + np.median(stairs_threshold)  # type: ignore
    return stairs_threshold.item()


def get_stairs(
    df: pd.DataFrame,
    stationary_threshold: float = 45,
    move_threshold: float = 0.1,
    run_threshold: float = 0.72,
    bicycle_threshold: float = 40,
    bouts_length: int = 5,
) -> tuple[pd.Series, float]:
    stairs_threshold = _get_stairs_threshold(df, run_threshold)

    valid = (
        (stairs_threshold < df["direction"])
```

```
        & (df["direction"] < bicycle_threshold)
        & (move_threshold < df["sd_x"])
        & (df["sd_x"] < run_threshold)
        & (df["inclination"] < stationary_threshold)
    )

    valid = get_filtered_activity(valid, bouts_length)
    valid.name = "stairs"

    return valid, stairs_threshold

import pandas as pd

from ....utils import get_filtered_activity


def get_walk(
    df: pd.DataFrame,
    stairs_threshold: float,
    stationary_threshold: float = 45,
    move_threshold: float = 0.1,
    run_threshold: float = 0.72,
    bouts_length: int = 2,
) -> pd.Series:
    valid = (
        (move_threshold < df["sd_x"])
        & (df["sd_x"] < run_threshold)
        & (df["direction"] < stairs_threshold)
        & (df["inclination"] < stationary_threshold)
    )

    valid = get_filtered_activity(valid, bouts_length)
    valid.name = "walk"

    return valid

import pandas as pd
from scipy.signal import medfilt

from ....utils import get_filtered_activity


def get_bicycle(
    df: pd.DataFrame,
    move_threshold: float = 0.1,
    bicycle_threshold: float = 40,
    bouts_length: int = 15,
) -> pd.Series:
    valid = (
        ((bicycle_threshold - 15) < df["direction"])
        & (df["inclination"] < 90)
        & (move_threshold < df["sd_x"])
    )

    valid = pd.Series(medfilt(valid.astype(int), 9), index=df.index)
    valid = valid & ((df["hl_ratio"] < 0.5) | (df["direction"] > bicycle_threshold))

    valid = get_filtered_activity(valid, bouts_length)
    valid.name = "bicycle"

    return valid

import numpy as np
import pandas as pd
from scipy.signal import medfilt
```

```python
def get_steps(df: pd.DataFrame) -> pd.Series:
    df = df[["activity", "walk_feature", "run_feature"]].copy()
    system_sf = 30
    scale = system_sf / 2 * np.linspace(0, 1, 256)

    df["steps"] = 0
    df.loc[df["activity"].isin(["walk", "stairs"]), "steps"] = df["walk_feature"]
    df.loc[df["activity"] == "run", "steps"] = df["run_feature"]
    df["steps"] = scale[df["steps"]]
    df["steps"] = medfilt(df["steps"], 3)

    return df["steps"].astype(np.float32)

import numpy as np
import pandas as pd

from ...utils import get_angles


def get_reference_angle(
    df: pd.DataFrame,
) -> tuple[float, str]:
    x_threshold_lower = 0.1
    x_threshold_upper = (
        0.7  # NOTE: Originally 0.7. To match the walk.py, 0.72 should be used.
    )
    inclination_threshold = 45  # NOTE: Same as stationary_threshold in walk.py
    direction_threshold = 10

    angle_mdn_coefficient = 6
    angle_threshold_lower = -28  # FIXME: In new code this is -30, original: -28
    angle_threshold_upper = 15
    angle_manual_set = -16

    walk = (
        (df["sd_x"].between(x_threshold_lower, x_threshold_upper, inclusive="neither"))
        & (df["inclination"] < inclination_threshold)
        & (df["direction"] < direction_threshold)
    )
    walk = df[walk]

    if not walk.empty:
        reference_angle = (
            np.median(walk["direction"]) - angle_mdn_coefficient
        ).item()  # Walk direction reference angle (median, degrees)

        reference_angle = (
            reference_angle * 0.725 - 5.569
        )  # Correction factor based on RAW data.

    # TODO: Iterative approach to find the reference angle based on the previous online version.
    else:
        reference_angle = None

    if (
        reference_angle is None
        or (reference_angle < angle_threshold_lower)
        or (reference_angle > angle_threshold_upper)
    ):
        reference_angle = angle_manual_set
        mode = "default"
    else:
        mode = "automatic"

    radians = np.radians(reference_angle).item()
```

```
    start, end = df.index[0], df.index[-1]

    # print(
    #     f"Reference angle for thigh: {radians:.3f} radians ({reference_angle:.3f} degrees).
    Based on data {start} to {end}. Calculation: {mode}."
    # )

    return np.float32(radians), mode


def _rotate_sd(df: pd.DataFrame, reference_angle: float) -> pd.DataFrame:
    system_sf = 30
    sin = np.sin(reference_angle)
    cos = np.cos(reference_angle)

    sq_sin = np.square(sin)
    sq_cos = np.square(cos)

    sq_x = np.square(df["x"])
    sq_z = np.square(df["z"])

    sd = pd.DataFrame(index=df.index)

    sd["terms_x"] = (
        (sq_sin * df["sq_sum_z"])
        + (sq_cos * df["sq_sum_x"])
        + (2 * system_sf * sq_x)
        + (2 * sin * df["x"] * df["sum_z"])
        + (-2 * sin * cos * df["sum_dot_xz"])
        + (-2 * cos * df["x"] * df["sum_x"])
    )
    sd.loc[sd["terms_x"] <= 0, "terms_x"] = 0
    sd["sd_x"] = np.sqrt(1 / (2 * system_sf - 1) * sd["terms_x"])

    sd["terms_z"] = (
        (sq_sin * df["sq_sum_x"])
        + (sq_cos * df["sq_sum_z"])
        + (2 * system_sf * sq_z)
        + (2 * sin * cos * df["sum_dot_xz"])
        + (-2 * sin * df["z"] * df["sum_x"])
        + (-2 * cos * df["z"] * df["sum_z"])
    )
    sd.loc[sd["terms_z"] <= 0, "terms_z"] = 0
    sd["sd_z"] = np.sqrt(1 / (2 * system_sf - 1) * sd["terms_z"])

    sd["sd_y"] = df["sd_y"]

    return sd[["sd_x", "sd_y", "sd_z"]].astype(np.float32)


def rotate_by_reference_angle(
    df: pd.DataFrame,
    reference_angle: float,
) -> pd.DataFrame:
    df = df.copy()

    reference_angle = np.float32(reference_angle)
    cos_angle = np.cos(reference_angle)
    sin_angle = np.sin(reference_angle)

    rotation_matrix = np.array(
        [
            [cos_angle, 0, sin_angle],
            [0, 1, 0],
            [-sin_angle, 0, cos_angle],
        ]
    )
```

```
    df[["x", "y", "z"]] = df[["x", "y", "z"]].dot(rotation_matrix).astype(np.float32)
    df[["sd_x", "sd_y", "sd_z"]] = _rotate_sd(
        df, reference_angle
    )  # TODO: Maybe not needed

    df[["euclidean_distance", "inclination", "side_tilt", "direction"]] = get_angles(df)

    return df

import numpy as np
import pandas as pd


def _fix_inclination(df: pd.DataFrame) -> None:
    df["euclidean_distance"] = np.linalg.norm(df[["x", "y", "z"]], axis=1)
    df.loc[df["euclidean_distance"] == 0, "euclidean_distance"] = np.nan

    df["inclination"] = np.arccos(
        df["x"] / df["euclidean_distance"]
    )  # (0-180 degrees), based on x-axis

    df["inclination"] = np.degrees(df["inclination"])

    df[["euclidean_distance", "inclination"]] = df[
        ["euclidean_distance", "inclination"]
    ].astype(np.float32)


def rotate_by_90_degrees_over_x(df: pd.DataFrame) -> pd.DataFrame:
    # FIXME: This needs to probably rotate more then just those axes but also all the other
    features

    raise (
        NotImplementedError(
            "This function doesn't work correctly, fix before using it."
        )
    )

    # df = df.copy()
    # y_new = -df["z"]
    # z_new = df["y"]

    # df["y"] = y_new
    # df["z"] = z_new

    theta = -np.pi / 2  # 90 degrees

    # Rotation matrix around x-axis
    rotation_matrix_x = np.array(
        [
            [1, 0, 0],
            [0, np.cos(theta), -np.sin(theta)],
            [0, np.sin(theta), np.cos(theta)],
        ]
    )

    df[["x", "y", "z"]] = df[["x", "y", "z"]].dot(rotation_matrix_x).astype(np.float32)

    # fix the other features: sum_y, sum_z, sum_dot_xz, direction, side_tilt

    return df


def check_upside_down_flip(
    df: pd.DataFrame,
) -> tuple[bool, float]:
```

```
    valid_points = df[~df["inclination"].between(45, 135)]

    if valid_points.empty:
        print("Not enough data to check upside down flip. Skipping.")
        return False, None

    mdn = np.median(valid_points["x"]).item()
    flip = True if mdn < 0 else False

    return flip, mdn


def check_inside_out_flip_thigh(
    df: pd.DataFrame,
) -> tuple[bool, float]:
    system_hz = 30
    rows_per_hour = (system_hz * 2) * 60
    window = rows_per_hour * 3
    step = rows_per_hour
    min_periods = rows_per_hour

    valid_windows = (
        df["inclination"]
        .rolling(window=window, step=step, min_periods=min_periods)
        .apply(lambda x: np.percentile(x, 2) <= 45)
    ).astype(
        bool
    )  # Looping through the data to check if the angle is less than 45 degrees in specified
    window.
    valid_points = pd.Series(
        df.index.map(valid_windows), index=df.index, dtype="boolean"
    ).ffill()  # Fill forward to get the valid points, not just the windows.
    valid_points = valid_points[valid_points].index  # Get the valid points only
    valid_points = df.loc[df.index.isin(valid_points) & (df["inclination"] > 45)]

    if valid_points.empty:
        print("Not enough data to check inside out flip. Skipping.")
        return False, None

    mdn = np.median(valid_points["z"]).item()
    flip = True if mdn > 0 else False

    return flip, mdn


def check_inside_out_flip_trunk(df: pd.DataFrame) -> tuple[bool, float]:
    # valid_points = df.loc[df["inclination"] > 30]
    valid_points = df[~df["inclination"].between(45, 135)]  # standing

    if valid_points.empty:
        print("Not enough data to check inside out flip. Skipping.")
        return False, None

    mdn = np.median(valid_points["direction"]).item()
    flip = False if mdn > 0 else True

    return flip, mdn


def check_inside_out_flip(
    df: pd.DataFrame,
    placement: str,
) -> tuple[bool, float | None]:
    if placement == "trunk":
        flip, mdn = check_inside_out_flip_trunk(df)
    else:
        flip, mdn = check_inside_out_flip_thigh(df)
```

```
    return flip, mdn


def check_flipped_other(df: pd.DataFrame) -> pd.DataFrame:
    upside_down_flip, upside_down_mdn = check_upside_down_flip(df)

    if upside_down_flip:
        df[["x", "y", "side_tilt"]] = -df[["x", "y", "side_tilt"]]
        _fix_inclination(df)

        print(
            f"Flipped upside down ({upside_down_mdn:.3f}). Axes flipped: x, y, inclination,
    side_tilt."
        )

    return df


def fix_flipped_sensor(
    df: pd.DataFrame,
    placement: str,
) -> pd.DataFrame:
    df = df.copy()

    # TODO: Polish flipping functions
    if placement in ["arm", "calf"]:
        df = check_flipped_other(df)
        return df
    elif placement not in ["trunk", "thigh"]:
        raise ValueError(f"Unknown placement: {placement}")

    inside_out_flip, inside_out_mdn = check_inside_out_flip(
        df,
        placement,
    )
    upside_down_flip, upside_down_mdn = check_upside_down_flip(df)

    if inside_out_flip and upside_down_flip:
        columns = ["x", "z", "sum_x", "sum_z", "sum_dot_xz", "direction"]
        df[columns] = -df[columns]
        _fix_inclination(df)

        print(
            f"Flipped inside out ({inside_out_mdn:.3f}) and upside down ({upside_down_mdn:.3f}).
    Axes flipped: x, z, sum_x, sum_z, sum_dot_xz, direction, inclination."
        )
    elif inside_out_flip:
        columns = ["y", "z", "sum_y", "sum_z", "sum_dot_xz", "side_tilt", "direction"]
        df[columns] = -df[columns]

        print(
            f"Flipped inside out ({inside_out_mdn:.3f}). Axes flipped: y, z, sum_y, sum_z,
    sum_dot_xz, side_tilt, direction."
        )
    elif upside_down_flip:
        columns = ["x", "y", "sum_x", "sum_y", "sum_dot_xz", "side_tilt"]
        df[columns] = -df[columns]
        _fix_inclination(df)

        print(
            f"Flipped upside down ({upside_down_mdn:.3f}). Axes flipped: x, y, sum_x, sum_y,
    sum_dot_xz, side_tilt, inclination."
        )

    return df
```

```python
import pandas as pd


def _fix_off_bouts(
    df: pd.DataFrame,
    short_off_bouts: int,
    long_off_bouts: int,
) -> None:
    df["bout_id"] = (df["non_wear"] != df["non_wear"].shift()).cumsum()
    no_worn_intervals = df[df["non_wear"]].groupby(df["bout_id"])

    for _, interval in no_worn_intervals:
        df.loc[interval.index, "non_wear"] = False

        if len(interval) > long_off_bouts:
            df.loc[interval.index, "non_wear"] = True

        elif len(interval) > short_off_bouts:
            first_row_index = df.index.get_loc(
                interval.index[0]
            )  # type: int # type: ignore
            index_1 = df.index[first_row_index - 15]
            index_2 = df.index[first_row_index - 12]
            max_value = df.loc[
                index_1:index_2,
                "sd_sum",
            ].max()

            if max_value > 0.5:
                df.loc[interval.index, "non_wear"] = True

    df.drop(columns="bout_id", inplace=True)


def _fix_on_bouts(
    df: pd.DataFrame,
    on_bouts: int,
) -> None:
    df["bout_id"] = (df["non_wear"] != df["non_wear"].shift()).cumsum()
    wear_intervals = (
        df[~df["non_wear"]].groupby(df["bout_id"]).filter(lambda x: len(x) < on_bouts)
    )
    df.loc[wear_intervals.index, "non_wear"] = True
    df.drop(columns="bout_id")


def _fix_off_bouts_angles(
    df: pd.DataFrame,
    long_off_bouts: int,
) -> None:
    degrees_tolerance = 5

    df["bout_id"] = (df["non_wear"] != df["non_wear"].shift()).cumsum()
    no_worn_intervals = df[df["non_wear"]].groupby(df["bout_id"])

    for _, interval in no_worn_intervals:
        df.loc[interval.index, "non_wear"] = False

        if len(interval) > long_off_bouts:
            df.loc[interval.index, "non_wear"] = True

        else:
            angles_mean = interval[["inclination", "side_tilt", "direction"]].mean(
                axis=0
            )

            rule_1 = (abs(angles_mean - [90, 0, 90]) < degrees_tolerance).all()
```

```python
            rule_2 = (abs(angles_mean - [90, 0, -90]) < degrees_tolerance).all()

            if rule_1 or rule_2:
                df.loc[interval.index, "non_wear"] = True

    df.drop(columns="bout_id", inplace=True)


def get_non_wear(
    df: pd.DataFrame,
    short_off_bouts: int = 600,
    long_off_bouts: int = 5_400,
    on_bouts: int = 60,
) -> pd.Series:
    df = df[["sd_x", "sd_y", "sd_z", "inclination", "side_tilt", "direction"]].copy()
    df["sd_mean"] = df[["sd_x", "sd_y", "sd_z"]].mean(axis=1)
    df["sd_sum"] = df[["sd_x", "sd_y", "sd_z"]].sum(axis=1)

    df["non_wear"] = df["sd_mean"] < 0.01
    _fix_off_bouts(df, short_off_bouts, long_off_bouts)
    _fix_on_bouts(df, on_bouts)
    _fix_off_bouts_angles(df, long_off_bouts)

    return df["non_wear"]


def get_intervals_split_by_non_wear(non_wear: pd.Series) -> pd.DataFrame:
    # TODO: This should be moved to utils and used for flipping as well!
    if not non_wear.any():
        return pd.DataFrame(
            {
                "start": [non_wear.index[0]],
                "end": [non_wear.index[-1]],
                "wear": True,
            }
        )

    non_wear = non_wear.to_frame()
    non_wear["segment"] = (
        non_wear["non_wear"].ne(non_wear["non_wear"].shift())
    ).cumsum()
    intervals = (
        non_wear.reset_index()
        .groupby("segment")
        .agg(
            start=("datetime", "first"),
            end=("datetime", "last"),
            wear=("non_wear", "first"),
        )
    )

    intervals["wear"] = ~intervals["wear"]

    return intervals.sort_values(by="start", ascending=True)

from datetime import datetime, timedelta
from typing import Any, Callable

import numpy as np
import pandas as pd
from pydantic import BaseModel, TypeAdapter, field_validator

from .non_wear import get_intervals_split_by_non_wear


class Angle(BaseModel):
    value: float | list[float]
```

```
    expires: datetime | str | None = None

    @field_validator("expires", mode="before")
    def validate_datetime(cls, value):
        return pd.to_datetime(value)

    def update_intervals(self, intervals: pd.DataFrame) -> pd.DataFrame:
        intervals = intervals.to_dict(orient="records")
        starting_interval = intervals[0]

        if not starting_interval["wear"] or self.expires < starting_interval["start"]:
            starting_interval["mode"] = "automatic"

        else:
            if self.expires and (self.expires < starting_interval["end"]):
                propagated_interval = {
                    "start": starting_interval["start"],
                    "end": self.expires,
                    "angle": self.value,
                    "expires": self.expires,
                    "mode": "propagated",
                }
                starting_interval["start"] = self.expires
                intervals.insert(0, propagated_interval)
            else:
                starting_interval["angle"] = self.value
                starting_interval["mode"] = "propagated"
                starting_interval["expires"] = self.expires

        return pd.DataFrame(intervals)


class Calibration(BaseModel):
    start: datetime | str
    end: datetime | str
    ttl: timedelta | str = None

    @field_validator("start", "end", mode="before")
    def validate_datetime(cls, value):
        return pd.to_datetime(value)

    @field_validator("ttl", mode="before")
    def validate_timedelta(cls, value):
        return pd.to_timedelta(value)

    @property
    def expires(self) -> datetime | None:
        return self.end + self.ttl if self.ttl else None

    def get_intervals(
        self, interval: pd.DataFrame, tolerance: int = 5
    ) -> dict[str, Any] | None:
        calibration_interval = interval[
            (interval.index >= self.start) & (interval.index <= self.end)
        ]
        available_duration = len(calibration_interval)
        duration = (self.end - self.start).total_seconds()
        valid = np.isclose(duration, available_duration, atol=tolerance)

        if valid:
            if self.expires and self.expires < interval.index[-1]:
                end = self.expires
            else:
                end = interval.index[-1]

            if self.start > interval.index[0]:
                start = self.start
```

```python
            else:
                start = interval.index[0]

            return {
                "start": start,
                "end": end,
                "calibration_start": self.start,
                "calibration_end": self.end,
                "expires": self.expires,
                "mode": "calibration",
            }


def fix_overlapping_calibrations(intervals: list[dict[str, Any]]) -> None:
    for index, interval in enumerate(intervals):
        next_interval = intervals[index + 1] if index + 1 < len(intervals) else None

        if next_interval:
            if interval["end"] > next_interval["start"]:
                interval["end"] = next_interval["start"]


def fill_intervals(
    calibration_intervals: list[dict[str, Any]], df: pd.DataFrame
) -> pd.DataFrame:
    intervals = []
    current_end = df.index[0]

    for calibration in calibration_intervals:
        if current_end < calibration["start"]:
            intervals.append(
                {
                    "start": current_end,
                    "end": calibration["start"],
                    "mode": "automatic",
                }
            )

        current_end = calibration["end"]

    if current_end < df.index[-1]:
        intervals.append(
            {
                "start": current_end,
                "end": df.index[-1],
                "mode": "automatic",
            }
        )

    intervals += calibration_intervals

    return intervals


def get_updated_interval_by_calibrations(
    calibrations: list[dict[str, Any]], interval: pd.DataFrame
) -> pd.DataFrame | None:
    calibrations: list[Calibration] = TypeAdapter(list[Calibration]).validate_python(
        calibrations
    )  # Convert to list of Calibration objects
    calibrations = sorted(
        calibrations, key=lambda x: x.start
    )  # Sort by start time, otherwise it doesn't work.
    calibration_intervals = []

    for calibration in calibrations:
        calibration_interval = calibration.get_intervals(
```

```
            interval
        )  # Get the intervals for each calibration, only if calibration exists in the interval.
    Otherwise None.

        if calibration_interval:
            calibration_intervals.append(calibration_interval)

    if calibration_intervals:
        fix_overlapping_calibrations(
            calibration_intervals
        )  # Fix overlapping calibration intervals. If new calibration appears before the previous
     one expires.
        filled_intervals = fill_intervals(
            calibration_intervals, interval
        )  # Fill the gaps between the calibration intervals - create new intervals.
        return filled_intervals


def get_intervals(
    df: pd.DataFrame,
    non_wear: pd.Series,
    reference_angle: dict[str, Any],
    calibrations: list[dict[str, Any]],
) -> pd.DataFrame:
    final_intervals = []
    intervals = get_intervals_split_by_non_wear(
        non_wear
    )  # Split the dataframe by non-wear intervals.

    for interval in intervals[intervals["wear"]].itertuples():
        new_intervals = None

        if calibrations:
            new_intervals = get_updated_interval_by_calibrations(
                calibrations, df[interval.start : interval.end]
            )  # Get the calibration intervals for each interval, and fill the gaps between them.

        if new_intervals:
            final_intervals.extend(new_intervals)
        else:
            final_intervals.append(
                {
                    "start": interval.start,
                    "end": interval.end,
                    "mode": "automatic",
                }
            )

    final_intervals = (
        pd.DataFrame(final_intervals)
        .sort_values(by="start", ascending=True)
        .reset_index(drop=True)
    )

    final_intervals["wear"] = True
    intervals = (
        pd.concat([final_intervals, intervals[~intervals["wear"]]])
        .sort_values(by="start", ascending=True)
        .reset_index(drop=True)
    )
    intervals.loc[~intervals["wear"], "mode"] = "last_known"

    if reference_angle:
        angle = Angle(**reference_angle)

        if angle:
            intervals = angle.update_intervals(intervals)
```

```python
    return intervals


def calculate_angles(
    df: pd.DataFrame, intervals: pd.DataFrame, function: Callable
) -> pd.DataFrame:
    intervals = intervals.to_dict(orient="records")

    for interval in intervals:
        if interval["mode"] == "calibration":
            start = interval["calibration_start"]
            end = interval["calibration_end"]

        elif interval["mode"] == "automatic":
            start = interval["start"]
            end = interval["end"]
        else:
            continue

        temp = df.loc[start:end]
        interval["angle"], interval["calculation"] = function(temp)

    intervals = pd.DataFrame(intervals)
    intervals["angle"] = intervals["angle"].ffill()

    return intervals


def get_last_angle(intervals: pd.DataFrame) -> dict[str, Any]:
    angle = intervals.iloc[-1].to_dict()
    mode = angle.get("mode")
    calculation = angle.get("calculation")

    if (mode in ["propagated", "calibration"]) and calculation != "manual":
        return {
            "value": np.float32(angle["angle"]),
            "expires": angle["expires"],
        }
```
