# Supplementary material for: Validation and comparative study of the Motus system for accurately identifying movement behaviours using different sampling frequencies
Source: Sci Rep. 2025 Nov 27;15:42377. doi: 10.1038/s41598-025-26373-7 (PMC12661018; doi:10.1038/s41598-025-26373-7)
Supplement: Supplementary file 2 — Supplementary Material 2 [file 41598_2025_26373_MOESM2_ESM.docx]

## Additional file 2: Instrumentation details

*Table A1: Instrumentation-specific details for the accelerometer included in the present study.*

| **Attribute** | **SENSmotionPlus*** | **Axivity AX3*** |
| --- | --- | --- |
| **Sensing unit** | 3-axis accelerometer (X, Y, and Z) | 3-axis accelerometer (X, Y, and Z) |
| **Range** | ±4g | ±8 (adjustable: ±2-16g) |
| **Dimensions** | 47 x 22 x 4.5 mm | 7.6 x 23 x 32.5 mm |
| **Mass** | 7g | 11g |
| **Resolution** | 10 bit | Upto 13 bit |
| **Default Sample Frequency** | 12.5 Hz and 25 Hz | 100 Hz |
| **Manufacturer recommended position** | None specifically | None specifically |
| **Orientation Sensitive** | Yes | Yes |
| **Raw file** | HEX and .bin | .CWA (.csv available) |
| **Software** | Smartphone- and web application | Open Movement GUI v.1.0.0.30 |

**SENSmotionPlus is part of the Motus system, while Axivity AX3 is used as part of the ActiPASS tool.*
