# Supplementary material for: Validation and comparative study of the Motus system for accurately identifying movement behaviours using different sampling frequencies
Source: Sci Rep. 2025 Nov 27;15:42377. doi: 10.1038/s41598-025-26373-7 (PMC12661018; doi:10.1038/s41598-025-26373-7)
Supplement: Supplementary file 3 — Supplementary Material 3 [file 41598_2025_26373_MOESM3_ESM.docx]

## Additional file 4: Optimized algorithm for 12.5Hz

A correctional polynomial was added to the original Acti4 algorithm to accommodate the Sens 25Hz sensor, in order to approximate the standard deviation of an Axivity sensor. We extend this method to the 12.5Hz sensor, thus revising this correctional polynomial based on the ground truth (video observation from the present study). The correctional polynomial includes two parameters: $\alpha$ and $\beta$ and is defined as: $\hat{SD}=\alpha\cdot SD+\beta\cdot{SD}^{2}$. For the correctional polynomial fitted for SENS 25Hz the parameters are $\alpha=1.03$ and $\beta=0.18$. For SENS 12.5Hz we perform a grid search over these two parameters based on a range for $\alpha$ of 0.8 to 1.2 with an increment of 0.02, and a range for $\beta$ of 0 to 0.4 with an increment of 0.02. The data was split into training (70%) and test (30%).

The polynomial with the highest associated training F1-score was for the values $\alpha=1.14$ and $\beta=0.02$. The optimal solution was robust, with a very small gradient around the optimal parameter values. This indicates that many parameter values will result in practically identical models.

- Training F1-score: **0.947** (weighted by support) **0.946** (custom weights) **0.942** (macro)
- Test F1-score: **0.953** (weighted by support) **0.941** (custom weight**s) 0.943** (macro)


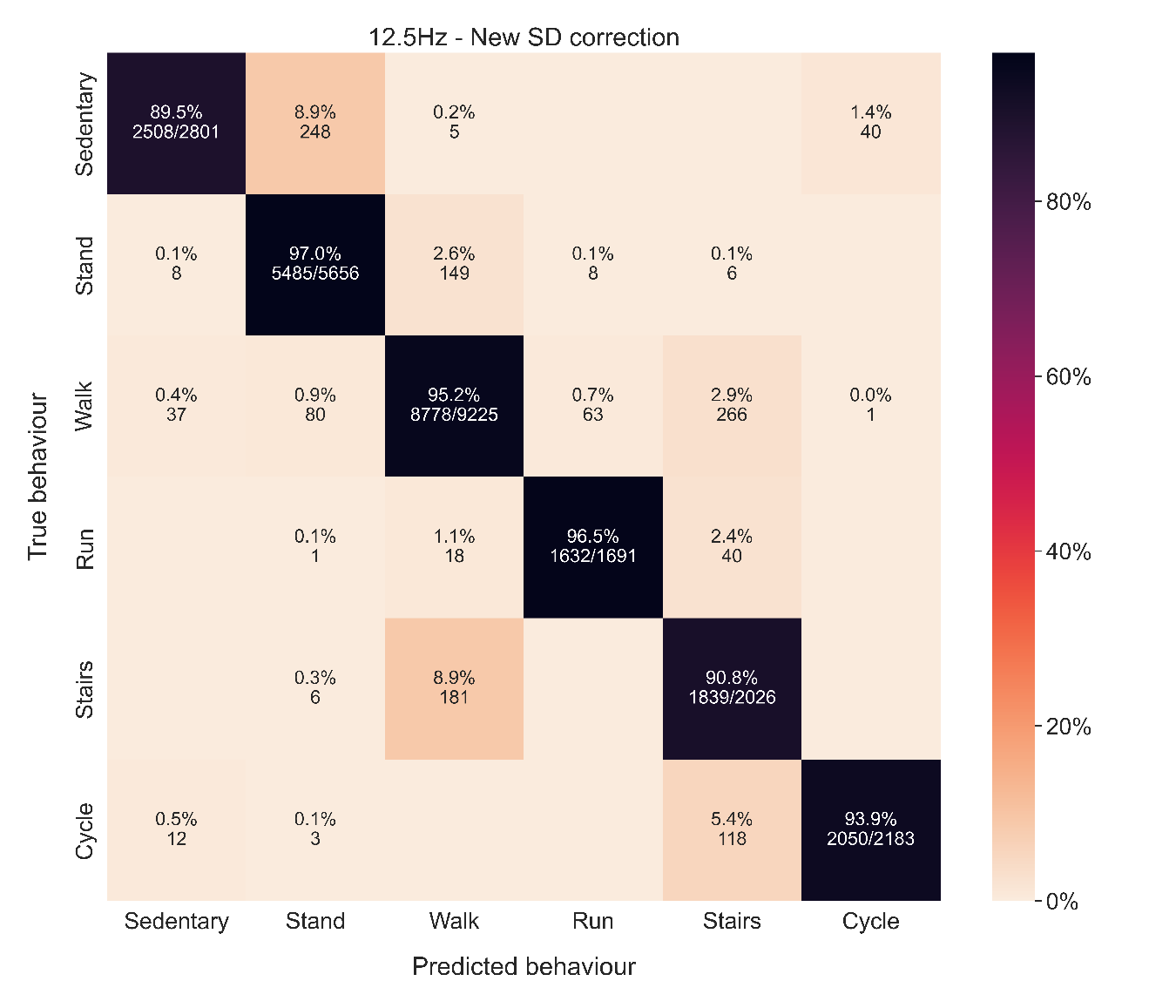
The performance of the new model is not significantly better than the baseline model. It performs slightly better for running, but slightly worse for stairs. However, the correction of the standard deviation feature is now based on the ground truth instead of another accelerometer. This adds, if not performance, validity to the algorithm when used with SENS accelerometers.

*Figure A1: Confusion matrices for the new SD-correction adjusted for SENSmotionPlus accelerometer 12.5 Hz.*

*The matrix compare the system's classification of six behaviours (sedentary, standing, walking, stairs, cycling) against video observations, based on the included sample (n=18). The Y-axis (rows) represents the true behaviour (based on video annotations), and the X-axis (columns) represents the predicted behaviour (from the accelerometer data). The diagonal elements, starting from the top-left to the bottom-right, represent correct classifications for each behaviour in % of correctly classified instances. Off-diagonal elements indicate misclassifications, where one behaviour was incorrectly classified as another. All annotated data is predicted for comparison with baseline model*.
