# Supplementary material for: Validation and comparative study of the Motus system for accurately identifying movement behaviours using different sampling frequencies
Source: Sci Rep. 2025 Nov 27;15:42377. doi: 10.1038/s41598-025-26373-7 (PMC12661018; doi:10.1038/s41598-025-26373-7)
Supplement: Supplementary file 4 — Supplementary Material 4 [file 41598_2025_26373_MOESM4_ESM.docx]

## Additional file 3: Movement behaviour definitions

Table A2: Description of movement behaviours included in the laboratory validation.

| **Movement behaviours** | **Descriptions*** |
| --- | --- |
| Sitting | When the person’s buttocks are on the seat of the chair, bed or floor. Sitting can include some movement in the upper body and legs; this should not be tagged as a separate transition. Adjustment of sitting position is allowed. |
| Standing | Upright, feet supporting the person’s body weight, with no feet movement. |
| Walking | Locomotion towards a destination with one stride or more, (one step with both feet, where one foot is placed at the other side of the other). Walking could occur in all directions. Walking along a curved line is allowed. |
| Stair | At least two steps in the same direction on a stair, either upstairs or downstairs.  Start: Heel-off of the foot that will land on the first step of the stairs.  End: When the heel-strike of the last foot is placed on flat ground.  If both feet rests at the same step with no feet movement, standing should be inferred. One step with both feet on flat surface (between) stairs, walking should be interfered. |
| Sit cycling (Stationary) | Pedalling while the buttocks is placed at the seat or standing up. Cycling starts on first pedalling and finishes when pedalling ends.  Not pedalling: Sitting or standing without pedalling should be tagged separate as sitting or standing. |
| Running | Locomotion towards a destination, with at least two steps where both feet leave the ground during each stride. |
| Undefined/non | Until all the sensors are attached, or final adjustment made to position the video camera can be tagged as undefined. All postures/movements that cannot be clearly identified due to blocking of the camera/view should be tagged as undefined. |
| Heel-drop | Tagged as the one frame before the heels hit the floor. The part between heel-drops is tagged as undefined. |

**The description guides the annotators in identifying and annotating the correct behaviour. The description is based on previous validation studies published on similar movement behaviours (5, 6, 39).*
